# Gene Expression in the Spinal Cord in Female Lewis Rats with Experimental Autoimmune Encephalomyelitis Induced with Myelin Basic Protein

**DOI:** 10.1371/journal.pone.0048555

**Published:** 2012-11-06

**Authors:** Hayley R. Inglis, Judith M. Greer, Pamela A. McCombe

**Affiliations:** University of Queensland Centre for Clinical Research, Brisbane, Queensland, Australia; University of Muenster, Germany

## Abstract

**Background:**

Experimental autoimmune encephalomyelitis **(**EAE), the best available model of multiple sclerosis, can be induced in different animal strains using immunization with central nervous system antigens. EAE is associated with inflammation and demyelination of the nervous system. Micro-array can be used to investigate gene expression and biological pathways that are altered during disease. There are few studies of the changes in gene expression in EAE, and these have mostly been done in a chronic mouse EAE model. EAE induced in the Lewis with myelin basic protein (MBP-EAE) is well characterised, making it an ideal candidate for the analysis of gene expression in this disease model.

**Methodology/Principal Findings:**

MBP-EAE was induced in female Lewis rats by inoculation with MBP and adjuvants. Total RNA was extracted from the spinal cords and used for micro-array analysis using AffimetrixGeneChip Rat Exon 1.0 ST Arrays. Gene expression in the spinal cords was compared between healthy female rats and female rats with MBP-EAE. Gene expression in the spinal cord of rats with MBP-EAE differed from that in the spinal cord of normal rats, and there was regulation of pathways involved with immune function and nervous system function. For selected genes the change in expression was confirmed with real-time PCR.

**Conclusions/Significance:**

EAE leads to modulation of gene expression in the spinal cord. We have identified the genes that are most significantly regulated in MBP-EAE in the Lewis rat and produced a profile of gene expression in the spinal cord at the peak of disease.

## Introduction

On the basis of pathological findings and genetic studies that show that the majority of genes that predispose to multiple sclerosis (MS) are associated with T cell activation [1], MS is thought to be primarily a T-cell mediated autoimmune disease. B cells and antibodies are also thought to play a role in pathogenesis [2]. The symptoms of MS are due to demyelination and axonal loss, and possibly to temporary loss of nerve conduction due to circulating factors [3]. Experimental autoimmune encephalomyelitis (EAE) is the best available animal model of MS, and can be induced by inoculation of susceptible animals with a range of central nervous system (CNS) antigens.

EAE was first induced by inoculation of whole brain tissue [4]; [5]. Myelin basic protein (MBP) was the first encephalitogenic protein to be studied in detail. Later studies have used myelin proteolipid protein (PLP) [6]; [7] and myelin oligodendrocyte glycoprotein (MOG) [8] to induce EAE. The susceptibility to EAE varies among animal strains and with the antigen used to induce the disease. There have been studies of the genetic loci that contribute to this susceptibility. The most important genes are the MHC class II, but other genes have been implicated, including other immune related genes [9]–[11]. Studies have also identified genetic loci that contribute to different EAE traits such as the severity of disease, maximum clinical score and the presence of demyelination [9]; [10].

In this study we have investigated MBP-EAE, which is characterized by weight loss and ascending paralysis [12] followed by spontaneous recovery. It has been clearly shown that recovery from weakness is associated with restoration of electrical conduction [13]. The pathology of MBP-EAE involves infiltration of the spinal cord and nerve roots with inflammatory cells [14], followed by demyelination of axons. In MBP-EAE there is apoptosis within the spinal cord [15]; [16] and there is also axonal damage [17]. The metabolic processes that cause damage in EAE include glutamate toxicity [18] and dysregulation of ion channels including calcium channels [19]. Because of the detailed knowledge of the pathogenesis of this EAE model, it is of interest to determine how gene expression correlates with the known features of this disease. The advantages of using EAE for studies of gene expression in the CNS are that tissue can be easily obtained at the peak of disease, without a significant post-mortem delay, allowing good preservation of RNA for expression studies.

Because of the extensive characterization of MPB-EAE in the Lewis rat, it is an excellent candidate for studies aimed at defining the gene expression profile of the disease. There have been no previous gene array studies of MBP-EAE in the Lewis rat; however, the expression profile of selected cytokines, chemokines, adhesion molecules, cell markers, matrix metalloproteinases and other genes relevant to disease etiology have been examined in this model by real-time PCR (RT-PCR) [20]. There have been micro-array studies of gene expression in MBP-EAE in SJL/J mice [21] and of the effects of estrogen treatment on gene expression in MBP-EAE in BV8S2/AV4 double transgenic mice [22]. There have also been microarray studies of EAE induced with myelin-oligodendrocyte glycoprotein (MOG) in the spinal cords of mice [23] and rats [24]. The present study was carried out to investigate changes in gene expression in MBP-EAE compared to control Lewis rats, and found that there were significant differences from controls.

## Results

### a) Differential Gene Expression in MBP Induced EAE in the Lewis Rat

**Figure 1:**
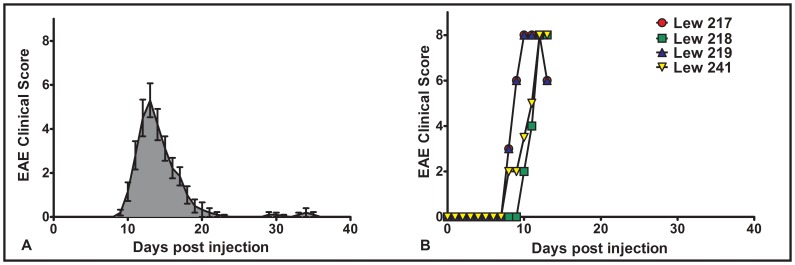
Clinical course of EAE. A. The figure **s**hows the results of a clinical course experiment (n  = 10) in which female Lewis rats (10–12 wks old) were injected with 50 µg Guinea Pig MBP emulsified in incomplete Freund’s Adjuvant containing 4 mg/ml heat inactivated *M.Butyricium*. The peak of disease generally occurs between 12 and 14 days post injection and resolves, usually within 7–10 days later. Mild relapses occur in a few animals between 28 and 40 dpi. **B.** Spinal cords used to prepare RNA for microarray analysis were collected on day 13 when the disease course was either at its peak or in the phase of very early resolution. Clinical course scores were recorded using a 12 point scale [43].

Rats were inoculated with MBP to induce EAE. The typical clinical course of this disease is shown in Figure 1A, which demonstrates that disease is generally monophasic with spontaneous recovery. Rats were sacrificed on day 13 post injection which corresponds approximately with the peak of clinical disease in our model. The course of disease of the rats that were used in the micro-array study is shown in Figure 1B.

**Figure 2:**
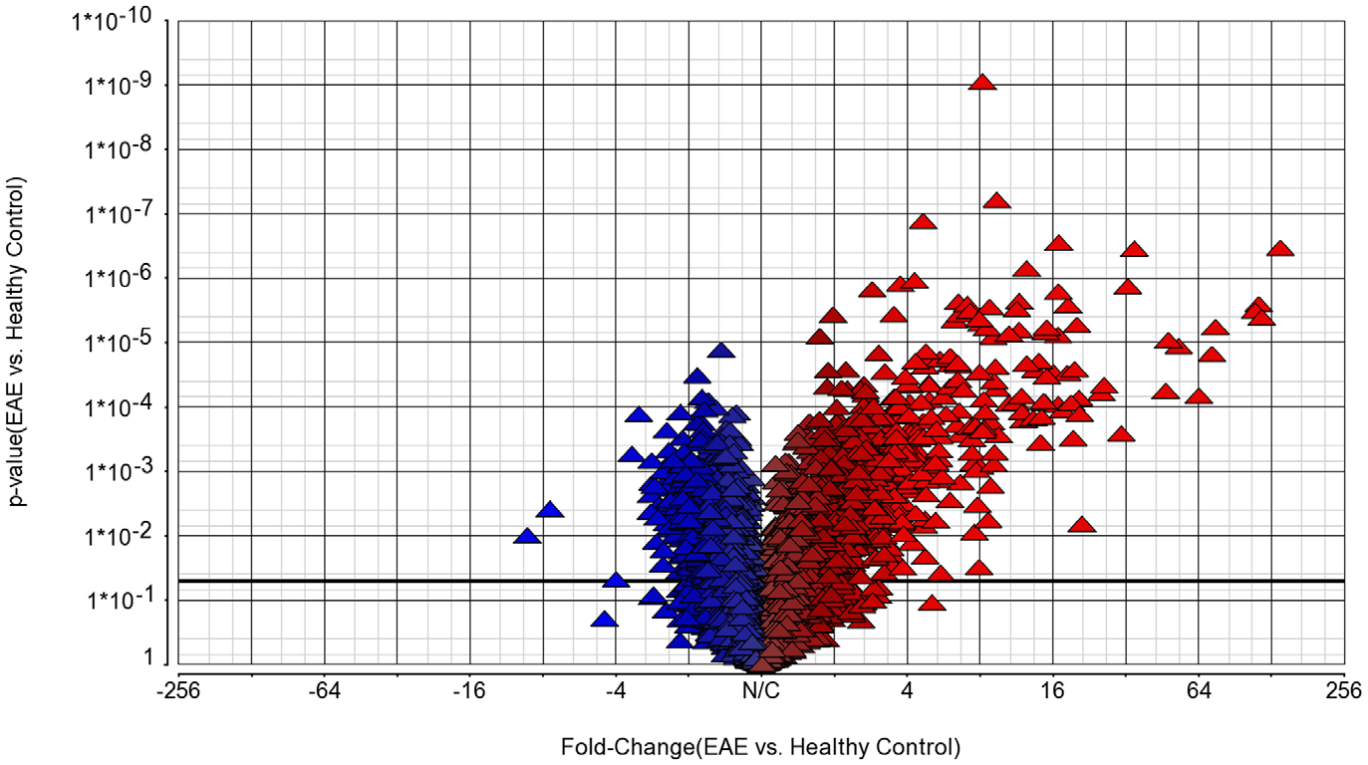
Volcano plot. This shows the significance (y axis) versus the fold change (x axis) for genes in rats with EAE compared to healthy controls.

RNA quality analysis was carried out on the BioRad Experion automated electrophoresis system. All preparations used in both assays had values of values of >9.5 for RNA quality indicator (RQI) [25]. This is illustrated in Figures S1 and S2. After array hybridization with the Affimetrix Rat Exon 1.0 ST array, which contains probe sets for 27,324 genes, data was uploaded for analysis with the Partek genomics suite. Transcripts from 8,793 genes were detected. Of these, we identified 2,350 genes that were significantly regulated between the healthy rats and rats with MBP-EAE (t test, (p-value <0.05). The overall gene expression changes in MBP induced EAE are illustrated in the volcano plot in Figure 2, which shows a large number of genes that were highly regulated compared to healthy controls.

Of the 2,350 differentially regulated genes, 2,265 mapped to known locations in the Partek data-base and the remainder were unmapped. Of these mapped genes, 1,665 were mapped to named genes. Of the 1,665 differentially regulated genes, 998 were found to be down-regulated and 667 were up-regulated. These are listed in Tables S1 and S2.

The 2,530 genes that were differentially regulated were up-loaded into the Ingenuity Pathway analysis software package (IPA) (Ingenuity ® Systems, www.ingenuity.com ) for functional and pathway analysis. After filtering to exclude genes that were not of relevance to the nervous system, we produced an annotated set of 1,190 differentially regulated genes. These genes were used for our further analysis of functions, pathways and upstream regulators.

To produce a list of the genes that are most significantly and highly regulated in MBP-EAE, we used a False Discovery Rate (FDR) [26] of 5% to further refine the data-set. This produced a list of the most significantly regulated genes (p values ≤0.0045). These are shown in Table 1, which lists the most significantly up-regulated genes (fold-change limit + or –4.0) and Table 2 which contains the most significantly down-regulated (fold change limit + or –2.0) transcripts in the data-set. A number of these genes mapped to loci that predispose to EAE in the rat (http://rgd.mcw.edu), or have been associated with one or more of the commonly used EAE models, as shown in Tables 1 and 2.

**Table 1:** Genes most significantly up-regulated at the peak of Clinical disease in MBP induced EAE in the Lewis Rat.

Entrez gene symbol	Entrez Gene Name	Fold change	p-value	QTL	Function	Previous association with EAE or Inflammation in the CNS	Ref
***Cd74***	CD74 molecule, major histocompatibility complex, class II invariant chain	139.3	3.35E–07	*EAEy EAEz*	Antigen processing and presentation	***Cd74*** maps to a gene regions associated with EAE in the DA rat.	[45]
***Psmb9***	proteasome (prosome, macropain) subunit, beta type, 9 (large multifunctional peptidase 2)	8.2	8.80E–10	*EAE1*	Antigen processing and presentation	***Psmb9*** maps to a gene region associated with EAE in the DA rat	[45]
***Rt1-a1***	rat major histocompatibility complex, class I	6.5	2.06E–05	–	Antigen processing and presentation	–	–
***Rt1-dmb***	rat major histocompatibility complex, class II, RTI-DM β chain	9.2	2.37E–05	–	Antigen processing and presentation	–	–
***Rt1-Ba***	rat major histocompatibility complex, class II, RT1-B α chain	32.7	1.33E–06	–	Antigen processing and presentation	***Rt1-Ba*** involved in antigen presentation in MBP induced EAE in the Lewis rat.	[46]; [47]
***Rt1-Da***	rat major histocompatibility complex, class II, RT1-D α chain	116.7	4.04E–06	–	Antigen processing and presentation	***Rt1-Da*** involved in antigen presentation in MBP induced EAE in the Lewis rat.	[46]; [47]
***Rt1-Db1***	rat major histocompatibility complex, class II, RT1-Dβ 1 chain	8.3	7.54E–05	–	Antigen processing and presentation	***Rt1-Db1*** involved in antigen presentation in MBP induced EAE in the Lewis rat.	[46]; [47]
***Tap1***	transporter 1, ATP-binding cassette, sub-family B (MDR/TAP)	4.3	1.08E–06	*EAE1*	Antigen processing and presentation	***Tap1*** maps to a gene region associated with EAE in the DA rat	[45]
***Timp1***	TIMP metallopeptidase inhibitor 1	8.0	2.86E–05		Antigen processing and presentation	***Timp1*** has been previously identified as up-regulated in mouse models of EAE.	[48]
***Casp1***	caspase 1, apoptosis-related cysteine peptidase (interleukin 1, beta, convertase)	6.3	4.48E–06	–	Apoptosis and cell death	***Caspase 1*** levels correlate with clinical disease course and the transcription rate of pro-inflammatory cytokines.	[32]; [33]
***Casp4***	caspase 4, apoptosis-related cysteine peptidase	7.1	2.52E–06	–	Apoptosis and cell death	Oligodendrocyte cell death in MOG induced EAE in mice was mediated by a pathway that involves ***Casp4.***	[49]
***Casp8***	caspase 8, apoptosis-related cysteine peptidase	4.3	1.93E–05	–	Apoptosis and cell death	***Casp8*** involved in neuronal cell death by apoptosis in EAE in the Lewis rat.	[50]
***Gda***	guanine deaminase	15.1	5.82E–06	–	Apoptosis and cell death	–	–
***Irf1***	interferon regulatory factor 1	8.6	5.89E–06	*EAE3 EAE18*	Apoptosis and cell death	***Irf1*** maps to a gene locus conferring resistance to MOG induced EAE in the rat.	[51]
***Irgm***	immunity-related GTPase family, M	14.0	1.93E–05	*EAE3 EAE18*	Apoptosis and cell death	***Irgm*** maps to a gene region conferring resistance to MOG induced EAE in the rat.	[51]
***Lcn2***	lipocalin 2	75.2	5.72E–06	–	Apoptosis and cell death	***Lcn2*** has previously been identified as upregulated in rat and mouse models of EAE.	[28]; [29]; [52]
***Tlr2***	toll-like receptor 2	6.0	4.77E–05	–	Apoptosis and cell death	***Tlr2*** is upregulated in active lesions of MS and MOG induced EAE in the DA rat.	[35]
***Tlr4***	Tlr4	4.9	8.16E–04	–	Apoptosis and cell death	***Tlr4*** is upregulated in active lesions of MS and MOG induced EAE in the DA rat.	[34]; [35]
***Trpm2***	transient receptor potential cation channel, subfamily M, member 2	4.6	8.25E–05	–	Apoptosis and cell death	–	–
***Ubd***	ubiquitin D	4.3	7.31E–04	–	Apoptosis and cell death	–	–
***Anxa3***	annexin A3	8.1	5.01E–06	–	Cellular movement	–	[52]
***Ccl7***	chemokine (C-C motif) ligand 7	6.7	2.65E–04	*EAE18b*	Cellular movement	***Ccl7*** maps to a gene region associated with MOG EAE in the rat	[53]
***Ccr5***	chemokine (C-C motif) receptor 5	6.5	2.25E–05	–	Cellular movement	***Ccr5*** was upregulated in the spinal cords of rats with MOG induced EAE	[54]
***Cx3cr1***	chemokine (C-X3-C motif) receptor 1	5.6	1.19E–03	–	Cellular movement	***Cx3cr1*** is involved in neuroinflammation and upregulated in phagocytic cells in the rat brain in MOG induced EAE.	[55]; [56]
***Cxcl9***	chemokine (C-X-C motif) ligand 9	113.50	2.51E–06	–	Cellular movement	***Cxc19*** is involved in neuroinflammation.	[55]
***Cxcl16***	chemokine (C-X-C motif) ligand 16	20.1	5.28E–06	*EAE18 EAE18a*	Cellular movement	***Cxcl16*** maps to gene region previously associated with a number of inflammatory diseases including MOG induced EAE in the DA rat.	[53];
***Fn1***	fibronectin 1	5.3	1.35E–03	–	Cellular movement	–	–
***Apobec1***	apolipoprotein B mRNA editing enzyme, catalytic polypeptide 1	46.8	5.61E–05	*EAE20, EAE22*	Cellular growth and proliferation	***Apobec1*** maps to gene regions previously associated with EAE in the mouse.	[48]
***Gpnmb***	glycoprotein (transmembrane) nmb	16.1	2.89E–05	–	Cellular growth and proliferation	–	–
***Grn***	Granulin	6.0	1.61E–05	–	Cellular growth and proliferation	***Grn*** enhances the proliferation of mouse neural progenitor cells in culture	[58]
***IL2ra***	interleukin 2 receptor, alpha	7.6	1.94E–04	–	Cellular growth and proliferation	MOG Induced EAE susceptible DA rats had higher ***IL-2Rα***, expression in naïve T cells compared to the resistant PVG rats.	[59]
***IL2rg***	interleukin 2 receptor, gamma	34.7	3.46E–07	–	Cellular growth and proliferation	Common cytokine receptor gamma-chain (gamma(c)) family cytokines have crucial roles in the development, proliferation, survival and differentiation of multiple cell lineages of both the innate and adaptive immune systems.	[60]
***Klrk1***	killer cell lectin-like receptor subfamily K, member 1	13.5	2.66E–05	–	Cellular growth and proliferation	–	–
***Laptm5***	lysosomal protein transmembrane 5	9.2	5.08E–04	–	Cellular growth and proliferation	–	–
***Stat1***	signal transducer and activator of transcription 1, 91 kDa	12.5	2.11E–05	–	Cellular growth and proliferation	Expression of ***Stat1*** increases to the recovery stage of MBP induced EAE in the Lewis rat.	[61]
***Irf8***	interferon regulatory factor 8	4.8	1.37E–05	–	Cellular development and differentiation	–	–
***Arhgdib***	rho GDP dissociation inhibitor (GDI) beta	8.4	1.18E–04	*EAE22*	Cell- cell signalling and interaction	***Arhgdib*** maps to gene region previously associated with a number of inflammatory diseases including MOG induced EAE in the DA rat.	[57]
***Emr1***	egf-like module containing, mucin-like, hormone receptor-like 1	16.9	1.60E–06	–	Cell- cell signalling and interaction	***–***	–
***Prkcd***	protein kinase C, delta	4.1	2.49E–04	–	Cell- cell signalling and interaction	***–***	–
***Ptafr***	platelet-activating factor receptor	6.5	3.74E–05	–	Cell- cell signalling and interaction	***–***	–
***Rac2***	ras-related C3 botulinum toxin substrate 2 (rho family, small GTP binding protein Rac2)	6.5	2.31E–06	–	Cell- cell signalling and interaction	***–***	–
***Tyrobp***	TYRO protein tyrosine kinase binding protein	7.3	2.02E–04	–	Cell- cell signalling and interaction	***Tyrobp*** maps to gene regions previously associated with EAE in the mouse.	[48]
***C3***	complement component 3	26.1	4.58E–05	–	Complement activation	***C3*** is required for full development of EAE in MOG induced EAE in mice	[62]
***C1qa***	complement component 1, q subcomponent, A chain	5.8	1.27E–04	–	Complement activation	The complement system has previously been implicated in the development of EAE, ***C1qa*** has been previously identified as up-regulated in mouse models of EAE.	[31]; [48]
***C1qb***	complement component 1, q subcomponent, B chain	4.6	1.06E–03	–	Complement activation	The complement system has previously been implicated in the development of EAE ***C1qb*** has been previously identified as up-regulated in mouse models of EAE	[31]; [48]
***C1qc***	complement component 1, q subcomponent, C chain	10.6	8.76E–05	–	Complement activation	The complement system has previously been implicated in the development of EAE ***C1qc*** has been previously identified as upregulated in mouse models of EAE	[31]; [48]
***C3ar1***	complement component 3a receptor 1	4.1	1.43E–03	*EAE20 EAE22*	Complement activation	***C3ar1*** maps to gene region previously associated with a number of inflammatory diseases including MOG induced EAE in the DA rat.	[57]
***Cfb***	complement factor B	4.7	2.89E–04	–	Complement activation	–	
***Serping1***	serpin peptidase inhibitor, clade G (C1 inhibitor), member 1	20.5	7.24E–05	–	Complement activation	–	–
***Soat1***	sterol O-acyltransferase 1	4.8	1.72E–05	*EAE17*	Complement activation	***Soat1*** maps to gene regions associated with MOG EAE in the rat,	[63]
***Btk***	Bruton agammaglobulinemia tyrosine kinase	5.5	1.80E–05	–	Inflammatory Response	–	–
***Cd4***	CD4 molecule	12.5	7.05E–07	*EAE20 EAE22*	Inflammatory Response	***Cd4*** maps to gene region previously associated with a number of inflammatory diseases including MOG induced EAE in the DA rat, CD4 expression on microglia correlates with resolution of disease in MBP induced EAE in the Lewis rat.	[57]; [64]
***Cd14***	CD14 molecule	4.4	4.44E–03	*EAEz*	Inflammatory Response	***Cd14*** maps to a gene region associated with EAE in the DA rat.	[45]
***Cd86***	CD86 molecule	10.5	7.23E–06	–	Inflammatory Response	***B7***/***CD86*** costimulation is critical for activation and expansion of MOG Fcgr2 areactive T cells in a mouse model of EAE.	[65]
***Chi3l1***	chitinase 3-like 1 (cartilage glycoprotein-39)	9.1	7.66E–04	*EAE14 EAE17*	Inflammatory Response	***Chi3l1*** maps to gene regions associated with MOG EAE in the rat, and has previously been identified as upregulated in mouse models of EAE.	[63]; [66] [48]
***Fcgr2a***	Fc fragment of IgG, low affinity IIa, receptor (CD32)	9.5	2.69E–04	*EAE17*	Inflammatory Response	***Fcgr2a*** maps to gene regions associated with MOG EAE in the rat.	[63]
***Fcgr2b***	Fc fragment of IgG, low affinity IIb, receptor (CD32)	11.6	6.35E–06	*EAE17*	Inflammatory Response	***Fcgr2b*** maps to gene regions associated with MOG EAE in the rat.	[63]
***F10***	coagulation factor X	5.4	4.64E–04	–	Inflammatory Response	–	–
***Il1b***	interleukin 1, beta	4.8	1.37E–03	–	Inflammatory Response	***Il1b*** is expressed by activated microglia in MOG induced EAE in the mouse.	[30]
***IL1rn***	interleukin 1 receptor antagonist	7.5	7.42E–04	–	Inflammatory Response	***IL1rn*** modulates a variety of interleukin 1 related immune and inflammatory responses.	[67]
***Lyn***	v-yes-1 Yamaguchi sarcoma viral related oncogene homolog	9.4	6.06E–08	–	Inflammatory Response	Severity of EAE is increased in ***lyn***−/− mice compared to WT controls.	[68]
***Ptprc***	protein tyrosine phosphatase, receptor type, C	17.0	2.78E–07	*EAE14 EAE17*	Inflammatory Response	***Ptprc*** maps to gene regions associated with MOG EAE in the rat.	[63]; [66]
***Vav1***	vav 1 guanine nucleotide exchange factor	4.0	4.36E–05	–	Inflammatory Response	***–***	–
***Pygl***	phosphorylase, glycogen, liver	8.0	4.28E–06	–	Other metabolic processes	–	–
***Cp***	ceruloplasmin (ferroxidase)	6.8	5.35E–05	–	Other metabolic processes	***CP*** Increases with oedema and has previously been identified as upregulated in mouse models of EAE	[48]; [69]

### b) Quantitative Real-Time PCR (rt PCR) Validation of Micro-array Data

**Figure 3:**
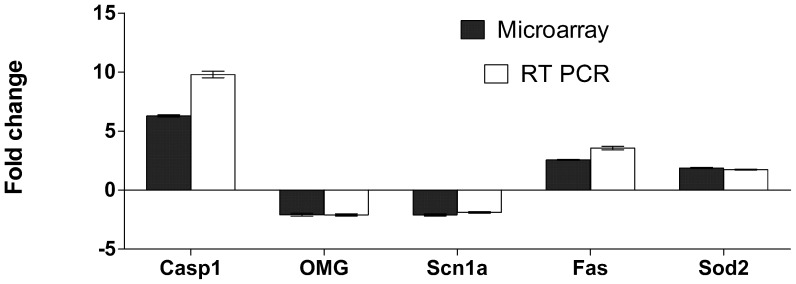
RT- PCR validation of 5 genes expressed differentially in the spinal cords of Lewis rats with MBP induced EAE. Tissue samples were snap frozen in liquid nitrogen and stored at −80°C prior to total RNA preparation using the QIAGEN RNeasy Lipid tissue kit. RNA quality analysis was carried out on the BioRadExperion automated electrophoresis system. All preparations used in both assays had RNA quality indicator (RQI) values of >9.5. For RT-PCR, total RNA was reverse transcribed and amplified as described in the methods. Analysis of selected genes up or down regulated at the peak of disease in EAE. Bars represent the average fold change between expression in the spinal cord level at peak of disease compared to normal healthy animals (+/− SEMs, Microarray n  = 4, RT-PCR n  = 8). Dark columns represent fold change derived from the microarray data. Similar amplification patterns were obtained from RT-PCR amplification of the same total RNA samples and a second set of 4 animals samples at an identical time point.

To confirm our micro-array findings, expression levels of a the genes caspase 1 (Casp1), oligodendrocyte myelin glycoprotein (OMG), alpha subunit voltage-gated sodium channel type 1 (Scn1a), Fas (TNF receptor superfamily member 6) and superoxide dismutase 2, mitochondrial (Sod2) were validated using RT-PCR in 8 rats in each group. These genes were selected as being regulated in EAE and of biological interest. Gene transcripts were amplified from cDNA preparations. These were obtained from the original total RNA samples used in the micro-array analysis and an additional 4 normal rats with MBP-EAE and an additional 4 healthy rats. The results are shown in Figure 3. All transcripts amplified showed similar expression patterns in the RT-PCR assay compared to the micro-array analysis.

**Table 2:** Genes most significantly down-regulated at the peak of Clinical disease in MBP induced EAE in the Lewis Rat.

Gene Symbol	Entrez Gene name	Fold change	p-value	QTL	Function	Previous association with EAE or Inflammation in the CNS	Ref
***Idi1***	isopentenyl-diphosphate delta isomerase 1	−3.4	5.34E–04	–	Cholesterol Biosynthesis	–	–
***Hmgcs1***	3-hydroxy-3-methylglutaryl-CoA synthase 1	−3.2	1.28E–04	–	Cholesterol Biosynthesis	–	–
***Cyp51a1***	cytochrome P450, family 51, subfamily A, polypeptide 1	−2.9	2.31E–03	–	Cholesterol Biosynthesis	Cytochhrome P450 dependent enzyme activities in the brain were reduced in both LPS induced and EAE models of inflammation in astrocytes in LPS induced inflammation.	[70]
***Msmo1***	methylsterol monooxygenase 1	−2.4	4.67E–04	–	Cholesterol Biosynthesis	–	–
***Sc5dl***	sterol-C5-desaturase	−2.2	1.07E–03	–	Cholesterol Biosynthesis	–	–
***Sqle***	squalene epoxidase	−2.1	2.58E–03	–	Cholesterol Biosynthesis	–	–
***Npy***	neuropeptide Y	−2.1	3.00E–04	*EAE25*	Cellular growth and proliferation	***Npy*** maps to a gene regipon that regulates EAE and suppresses EAE in the rat.	[71]; [72]
***Ptn***	pleiotrophin	−2.1	7.33E–04	–	Cellular growth and proliferation	***Ptn*** found to be up-regulated in recovery from EAE in the rat.	[73]
***Sept3***	septin 3	−2.1	5.28E–04	–	Cellular growth and proliferation	–	–
***Gjb1***	gap junction protein, beta 1	−2.0	1.08E–03	–	Cell-cell signalling and Interaction	***Gjb1*** (Connexin32) null mice develop demyelinating peripheral neuropathy	[74]
***Grb14***	growth factor receptor-bound protein 14	−2.1	5.55E–04	*Neuinf1*	Cell-cell signalling and Interaction	***Grb14*** maps to a gene region regulating the inflammatory microglial response in the rat.	[75]
***Hnmt***	histamine N-methyltransferase	−2.8	6.78E–04	–	Cell-cell signalling and Interaction	–	–
***Il1rapl1***	interleukin 1 receptor accessory protein-like 1	−2.5	2.29E–04	–	Cell-cell signalling and Interaction	–	–
***Nlgn1***	neuroligin 1	−2.0	9.21E–04	–	Cell-cell signalling and Interaction		–
***Ppp1r14c***	protein phosphatase 1, regulatory (inhibitor) subunit 14C	−2.2	1.74E–03	*Neuinf 4*	Cell-cell signalling and Interaction	***Ppp1r14c*** maps to a gene region regulating the inflammatory microglial response in the rat.	[75]
***Trhr***	thyrotropin-releasing hormone receptor	−2.5	1.82E–03	–	Cell-cell signalling and Interaction	–	–
***Ugt8***	UDP glycosyltransferase 8	−2.2	3.64E–03	–	Myelination	–	–
***Gal***	-galanin prepropeptide	−2.2	1.20E–04	*EAE6*	Inflammatory Response	***Gal*** maps to a gene region in the rat associated with models of rheumatoid arthritis and EAE.	[45]
***Galnt13***	**-**UDP-N-acetyl-alpha-D-galactosamine:polypeptide N-acetylgalactosaminyltransferase 13 (GalNAc-T13)	−2.8	1.66E–03	–	Post translational protein modification	–	–

### c) Biological Functions Most Significantly Regulated in MBP-EAE

The functional predictions that are generated from IPA are based on the direction of expression of a number of downstream genes which have been previously shown to be associated with that function. The functions that are most significantly altered in MBP-EAE are shown in Tables 3 and 4. The functions that are predicted to be up-regulated include activation of central nervous system cells, cell death or apoptosis (of leukocytes, microglia and dopaminergic neurons), formation of amyloid fibrils, and proliferation of oligodendrocyte precursor cells. The functions that are predicted to be down-regulated include long term-potentiation, neurotransmission, synaptic transmission, quantity of vesicles and delay in the death of neurons.

**Table 3:** Cellular functions significantly up-regulated in MBP induced EAE in the Lewis rat.

Function	Prediction	No of genes in direction of prediction	Z score	Overlap p-value	Genes for which expression direction predicts an increase in function*	Genes for which expression direction predicts a decrease in function	Genes which affect the function
Activation of central nervous system cells	Increased	*9 of 12*	2.268	2.83E–03	***Il1b,Gfap, Ptgs2, Vim, Il1rn, Cebpb, Tlr2, Tlr4***, *Npc1*	*Kcnj10,Ace*	*Gjd2*
Activation of astrocytes	Increased	*9 of 10*	*2.595*	*1.55E–03*	***Il1b,Gfap, Ptgs2, Vim, Il1rn, Cebpb, Tlr2, Tlr4***, *Npc1*	*Kcnj10,Ace*	
Apoptosis of leukocytes	Increased	*82 of 136*	*2.328*	*2.06–06*	***Tnfrsf1a, Fas, Irf1, Myd88, Lcn2, Tlr2, Tlr4, Stat1***	***Irgm***	***Casp3, Casp4***
Apoptosis of microglia	Increased	*7 of 9*	*2.570*	*1.08–06*	***Fas, Irf1, Myd88, Lcn2, Tlr2, Tlr4, Stat1***	***Casp3, Casp4***	
Cell death of dopaminergic neurons	Increased	*10 of 13*	*2.071*	*4.94E–04*	*Park2, Ppargc1a*, TRB@, ***Cd4, Casp7, Casp8*** **,** *Lep* **, ** ***Casp3,*** * Shh, Mapk8ip1,*	*Snca* **, ** ***Cybb*** **,**	***Fas***
Formation of amyloid fibrils	Increased	*5 of 6*	*2.213*	*4.55E–03*	***Tnfrsf1a*** **,** *Mme*, ***Apoe,*** * Apba2*, ***Sod2***		***Psen1***
Proliferation of oligodendrocyte precursor cells	Increased	*5 of 5*	*2.236*	*7.83E–03*	***Cxcl2, Rps6kb2*** **,** *Thrb, * ***Igf1*** **,** *Plp1*		

* **Gene abbreviations are given in Tables S1 and S2.** Gene names in **Bold** indicate increased expression in EAE, normal italic font indicates decreased expression.

### d) Canonical Pathways Regulated in MBP-EAE

A number of Canonical pathways were identified in IPA as being significantly regulated in MBP-EAE, based on the expression profile of genes in MBP treated animals compared to healthy controls. These are shown in Figure 4. These include immune related pathways and neural pathways. The details of some of the pathways that were regulated are shown in Figures S3, S4, S5, S6, S7, S8, S9, S10, S11, S12, S13, S14, S15, S16, S17, S18, S19, S20.

**Table 4:** Cellular Functions significantly down-regulated in MBP induced EAE in the Lewis rat.

Function	Prediction	No of genes in direction of prediction	Z score	Overlap p value	Genes for which expression direction predicts a decrease in function*	Genes for which expression direction predicts an increase in function	Genes which affect the function
Long term-potentiation	Decreased	*28 of 43*	−*2.397*	*2.49E–06*	*Chrnb2, Rhob, Gnaq*, ***Il1b***, ***Ptk2b***, *Grin1,* ***Itpr1*** *, Kcna4, Reln, Vldlr,* ***ApoE***, *Grin2a, Grik1, Snca, Gucy1a3, Ppp1r1a, Rab3a,* ***B2m*** *, * ***Psen1***, *Grik2, Rims1, Chrna7,* ***Tap1*** **,** *Ppp1r1b, Cplx2, Pcdh8, Jph4, Adcy8*	*Ppp3r1, Gria2, Nrg1, Pcp4*, *Mgll*, ***Cybb*** *, * ***Il1rn*** *, Erbb4*, *Gal, Dlg4, Gria3*, ***Casp1***, *Omg*, *Ncf1*	***Tlr4***
Neurotransmission	Decreased	*13 of 26*	−*2.043*	*4.79E–03*	*Park2* ***, Il1b***, *Grin1*,*Grik1,Grm5*, ***Psen1***, *Grik2, Htr1a,Gria3,* ***Snap29*** **,** *Cacna1b, Slc8a3, Nlgn1*	*Gria2*, *Gal,*	*Chrna7*, *Chrm3,* ***Nqo1***, *Rab3a, Slc5a7, Snca*, ***Apo1***, *Chrna3, Chrnb2*
Synaptic transmission	Decreased	*10 of 12*	−*2.578*	*2.45E–02*	*Grin1,Grik1, Grm5*, ***Psen1***, *Grik2, Htr1a, Gria3,* ***Snap29*** **,** *Slc8a3*, *Nlgn1*	*Gria2, Gal*,	*Chrna7, Chrm3*, ***Nqo1***, *Rab3a, Slc5a7, Scna*, ***Apoe***, *Chrna3, Chrnb2*
Quantity of vesicles	Decreased	*7 of 8*	−*2.137*	*7.33E –03*	***Grn***, *Snca*, ***Bm2***, *Ptprn, Rims1*, ***Tap1***, *Syn1*,	***Abc1a***	
Quantity of dendritic spines	Decreased	*7 of 9*	*score -2.044*	*3.08E–03*	*Rhob, Grin1*, ***Gda***, *Pfn2, Il1rapl1*, ***Lcn2***, *Akap5,*	***Mark2***	*Arhgef7*
Size of axons	Decreased	*4 of 4*	−*2.000*	*1.24E–04*	*Nefm, Sod1, Nefh, Nefl*		
Delay in the death of neurons	Decreased	*4 of 4*	−*2.000*	*4.56E–02*	***Tp53*** **, ** ***Casp2, Casp3*** **, ** ***Bak1***		

* **Gene abbreviations are given in Tables S1 and S2.**Gene names in **Bold** indicate increased expression in EAE, normal italic font indicates decreased expression.

**Figure 4:**
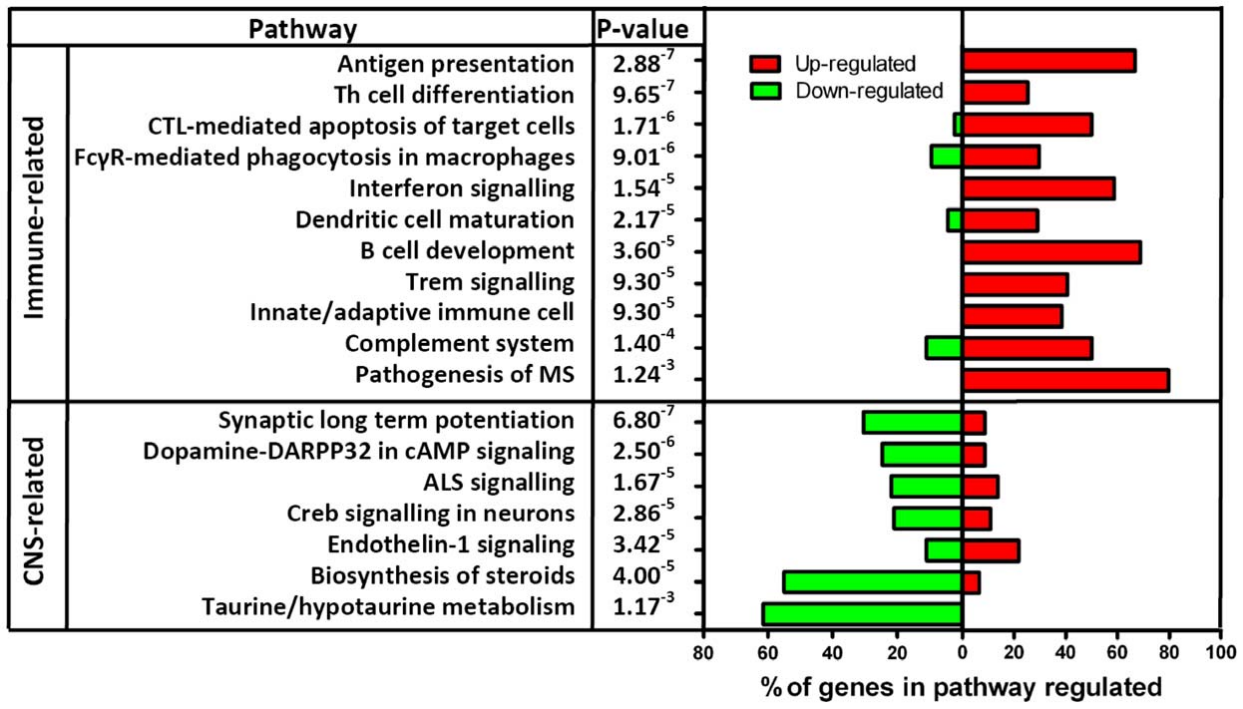
Summary of Canonical pathways that are regulated in MBP-EAE. The most regulated canonical pathways are listed. The percentage of genes of each specific pathway that are up-regulated are shown in red and the percentage of genes that are down-regulated are shown in green. The genes involved in each pathway are shown in individual supplementary figures.

### e) Upstream Factors Predicted to be Regulated in MBP-EAE

From IPA we have identified the upstream regulators that are predicted to be activated or inactivated in MBP-EAE. These are shown in Table 5. These include Sirtuin 2, stat1 and presenilin 2.

## Discussion

In this study, using female Lewis rats, we have investigated the expression of genes in MBP-EAE compared to controls. We have studied rats at day 13, which is the peak of clinical disease, when processes that lead to recovery are under way. The pathology of MBP-EAE involves infiltration of the CNS with macrophages and activated T lymphocytes [27]. There are morphological changes in microglia and astrocytes and there is demyelination of axons and axonal transaction. Consistent with this, we have found that MBP-EAE is associated with changes in expression of genes and biological functions of the immune system and also of the nervous system. We found markedly increased expression of MHC class II molecules and other immune related genes. This is consistent with the pathological findings of upregulation of MHC class II molecules and infiltration with immune cells [14]. Micro-array analysis of C57/BL6 mice with EAE induced with myelin-oligodendrocyte protein also found upregulation of numerous genes [23] including MHC class II and immune related genes. We note increased expression of lipocalin 2, which is a protein that can be involved in the immune response and that has been found to be upregulated in MOG-EAE in DA rats [28] and in C57/BL mice [29].

Genes involved in the inflammatory response and in antigen processing and presentation were some of the most highly regulated genes identified. Cell-cell signalling and interaction and cell movement were also highly represented amongst the most differentially regulated gene transcripts identified. A number of chemokines and their receptors, annexin 3A and fibronectin were all up-regulated more than four-fold in the diseased spinal cords. Cellular growth factors, notably IL2-α and the common gamma chain were highly up-regulated as was the inflammatory cytokine IL1-β which is expressed in activated microglial cells [30]. IL18, the IL1 receptor agonist IL1rn, the interleukin receptors IL1r1, IL4r, IL6r, and the interferon gamma inducible cytokine IL18 were all moderately up-regulated (Table S1).

The regulation of key components of the classical (C1q, C4, C3b, Serping1) and alternative (C3, Cfb) complement pathways and the complement component 3a receptor 1 (3ar1), but not molecules in the terminal pathway, is in accordance with previous studies which indicate that the common terminal pathway (culminating in the membrane attack complex) is not important for the development of fulminant demyelinating EAE [31].

Genes associated with apoptosis which were highly regulated in our data set include the apoptosis related serine peptidases (Caspases) which have all been associated with disease in various EAE models [32]; [33] and the toll like receptors 2 and 4 (Tlr2, Tlr4) which have previously been found up-regulated in active lesions of MS and MOG induced EAE in the DA rat [34]; [35]. As mentioned above, the apoptotic inducer lipocalin 2 (Lcn2) [36] which has previously been identified as up-regulated in MOG induced EAE in DA rats [28] and C57BL mice [29] was also highly expressed in MBP-EAE in the present study.

Down-regulation of gene transcripts in MBP induced EAE in the Lewis rat was much more subtle than up-regulation however almost a thousand genes were found to be significantly down-regulated in the model (Table S2). Eighteen of these were down-regulated more than 2-fold, notably including key enzymes in the cholesterol biosynthesis pathway and molecules involved in cellular growth and proliferation, signalling and interaction.

Of the most highly regulated genes in the spinal cord (Tables 1 & 2), many are associated with processes associated with induction of disease, such as cell-cell signalling, chemotaxis antigen presentation, activation of both infiltrating cells such as T-lymphocytes and macrophages and resident astrocytes and microglia. There is also regulation of genes associated with resolution of disease, for example apoptosis of immune infiltrating cells and activated glial cells, and growth and proliferation of oligodendrocyte precursor cells which migrate to the sites of demyelination and differentiate into mature oligodendrocytes able to carry out the remyelination of axonal processes damaged during the inflammatory phase of the disease [37].

**Table 5:** Upstream Regulators.

Gene ID	Gene name	Molecular Type	Predicted Activation State	Fold Change	Activation z-score	p-value of overlap	Gene regulation consistent with activation (Number of genes regulated of all potential gene targets)*	Genes regulation consistent with inhibition (Number of genes regulated of all potential gene targets)
***Sirt2***	Sirtuin2	Transcription regulator	Inhibited	–	−3.317	3.85E–09		*Sqle, Sc5dl, Pmvk, Mvd, Lss,Idi1, Hmgcr, Fnta, Fdft1, Dhcr7,Aacs(11 of 11)*
***IL1b***	interleukin 1, beta	cytokine	Activated	4.8	2.422	6.34E–06	***Vim, Sod2, Serpina3, Ptgs2, Psen1, Plau, Il1rn, Il1b, Igf1,Icam1, Fas, Cxcl2, Csf1, Casp4*** * (14 of 18)*	*Tac1, Snca, * ***Slc1a3*** *, Lep (4 of 18)*
***Mapt***	microtubule-associated protein tau	other	Activated	2.0		9.66E–05	*Pclo, Grin1, Dlgap2, Dlg4 (4 of 46)*	
***Crebbp***	CREB binding protein	Transcription regulator	Inhibited	–	−2.828	1.14E–04		*Grm1, Grin2d, Grin2c, Grin2a, Grin1, Gria3, Gria2, Camk1g*, (8 of 9)
***Stat1***	signal transducer and activator of transcription 1	Transcription regulator	Activated	12.5	2.197	2.27E–04	***Ubd, Stat2, Rsad2, Psmb9, Irf9, Irf8, Irf1, Icam1, Gbp2, Cxcl9, Ceacam1*** * (11 of 15)*	***Ubd, Stat2, Rsad2, Psmb9, Irf9, Irf8, Irf1, Icam1, Gbp2, Cxcl9, Ceacam1*** * (3 of 15)*
***Nfe2l2***	nuclear receptor subfamily 3, group C, member 1 (glucocorticoid receptor)	Transcription regulator	Inhibited	3.8	−2.125	4.04E–04	***Slc7a8, Ptpro, Nucb2,*** * Nqo1 (4 of 21)*	*Vsnl1, Syt5, Syn2, Slc30a4, Scg2, Reln, Pkia, Pafah1b, Kifc2, Hpca, Fgf13, Chgb, Calm1, Calb1 (14 of 21)*
***Tnf***	tumor necrosis factor	cytokine	Activated	–	3.575	7.32E–04	***Vcam1, Stat1, Sp1, Sod2, Ptgs2, Plau, Icam1, Fas, Csf1, Cflar, Cd14, Ccl7, Casp4*** * (13 0f 13)*	
***Tgfbr2***	transforming growth factor, beta receptor II (70/80 kDa)	kinase	Activated	3.1	2.137	1.61E–03	***Tgif1, Stat3*** *, Kcnn2, * ***Gfap, Fn1*** *, Chrm3, * ***Cd44, Cd14, Ccl7*** * (9 of 11)*	***Gja1*** *, Gal (2 of 11)*
***Nr3c1***	nuclear receptor subfamily 3, group C, member 1 (glucocorticoid receptor)	Ligand dependent nuclear receptor	Activated	–	2.626	3.36E–03	*Grm7,Grm5, Grm1, Grin2c, Gria4, Gria3, Gria2 (7 of 11)*	
***Tnfrsf1a***	tumor necrosis factor receptor superfamily, member 1A	Trans-membrane receptor	Activated	1.8	2.000	3.78E–03	***Sod2, P2rx7,*** * Dio2,Cflar (4 of 4)*	
***Parp1***	poly (ADP-ribose) polymerase 1	enzyme	Activated	1.6	2.429	9.30E–03	***Vcam1,Stat1, Sp1, Ptgs2, Il1b, Icam1*** * (6 of 6)*	
***Slc30a3***	solute carrier family 30 (zinc transporter), member 3	transporter	Inhibited	−1.3	−2.000	1.98E–02		*Snap25, Grin2a, Dlg4, Dcx (4 of 4)*
***Psen2***	presenilin 2	peptidase	Inhibited	–	−2.747	3.51E–02	*Gba2 (1 of 11)*	***Klra4*** *, Kcnab2, * ***Gja1, Gfap, Ctss, CD74, Cap1, C3ar1, C1qb, C1qa*** * (10 of 11)*

* **Gene abbreviations are given in Tables S1 and S2.** Gene names in ***Bold*** indicate increased expression in EAE, normal italic font indicates decreased expression.

EAE can only be induced in susceptible animals. In studies of different mouse and rat strains, some genetic loci have been identified as being important in the predisposition to EAE. In our study, many of the genes identified as being differentially regulated found to map to those regions (http://rgd.mcw.edu/).

From IPA, the cellular functions that are regulated can be predicted. The upregulated functions in MBP-EAE included activation of CNS cells, cell death or apoptosis (of immune cells, microglia and dopaminergic neurons), formation of amyloid fibrils, and proliferation of oligodendrocyte precursor cells. Apoptosis is known to be an important function in EAE. Apoptosis of T cells is important in the regulation of MBP-EAE [15]; [16]. Apoptosis of oligodendrocytes could also contribute to pathology in EAE. Consistent with this, we have found upregulation of genes associated with apoptosis. Our finding of increased proliferation of oligodendrocyte precursor cells is consistent with the observation that recovery in MBP-EAE is due to restoration of conduction in demyelinated fibres.

The functions that are predicted to be downregulated include long term-potentiation, neurotransmission, synaptic transmission, quantity of vesicles and delay in the death of neurons. These downregulated functions presumably reflect damage to neural tissue.

From IPA we also obtained a list of the canonical pathways that were regulated in MBP-EAE. There were changes in immune pathways and neural pathways. The immune related pathways include antigen presentation, T cell differentiation and complement pathways, all of which would be expected to be activated in EAE. The neural related pathways include dopamine feedback of cAMP signalling, Creb signalling in neurones, biosynthesis of steroids and ALS signalling.

From IPA we have identified the upstream regulators that are predicted to be activated or inactivated in MBP-EAE. The greatest inactivation was predicted in sirtuin 2 (sirt2). Sirt 2 is is a member of the sirtuin family of histone deacetylases and has been shown to have a role in promoting longevity [38]. With proteomic studies, sirtuin 2 has also been found to be downregulated in EAE [39]. Resveratrol which is an agonist of sirt2 has been shown to reduce the severity of EAE [40]. Stat1 is a member of the JAK/STAT group of proteins [41]. Stat 1 regulates genes involved in interferon signalling which is shown in this study to be upregulated in MBP-EAE. Presenilin 2 is a member of the gamma-secretase family and is predominantly expressed in neurones where it plays a role in the processing of amyloid precursor protein [42] and was predicted in this study to be downregulated.

In conclusion, we have shown that MBP induced EAE alters gene expression in the spinal cord. We have produced a gene expression profile for this model in the Lewis rat which correlates well with the documented characteristics of the disease. Further examination of the pathways and functions regulated should pave the way for future studies into their regulation which may lead to the identification of potential drug targets for enhancement of the resolution of inflammatory disease in the CNS.

## Materials and Methods

### Ethics Statement

This research was approved by the Animal Ethics Committee of the University of Queensland. The approval number was UQCCR/770/08/MSRA.

### Induction of EAE

EAE was induced in 11 wk old female Lewis rats by subcutaneous injection at the base of the tail of 50 µg recombinant MBP, using our standard methods (McCombe et al., 1996). Healthy age-matched unimmunized rats were used as controls. Weakness of the tail, hindlimbs and forelimbs was assessed by grading the degree of weakness of each region separately on a scale of 0 (no weakness) to 4 (total paralysis), as described previously [43]. On each day of examination, the scores from each region were added together to give a total clinical score per rat (maximum total clinical score = 12). Figure 1 shows the typical clinical course of rats with this form of EAE.

### Tissue Collection

On day 13 after inoculation, which is the peak of clinical disease, rats were sacrificed and the spinal cord was excised from the spinal column by insufflation using a large bore blunt ended needle and a 60 ml syringe. Figure 1B shows the clinical course of these rats up to the day of sacrifice. Tissue was snap frozen in liquid nitrogen prior to being stored at −80°C. Four rats in each group were used for micro-array analysis and a total of 8 rats in each group were used for rt-PCR.

### RNA Extraction

Total RNA was extracted from tissues using QIAGEN RNeasy® Lipid tissue Midi kits as per the manufactures instructions and treated with DNase1 (QIAGEN) to remove all traces of genomic DNA prior to storage at −80°C. The RNA quality analysis was carried out using the BioRadExperion automated gel electrophoresis system (BioRad Laboratories Inc.).

### Array Hybridization

For each group, 4 biological replicates were used. cDNA synthesis and amplification was performed using the Applause WT-Amp Plus ST kit (NuGEN). Samples were enzymatically fragmented and biotinylated using the Encore Biotin Module labelling kit (NuGEN). Samples were hybridized to AffimetrixGeneChip Rat Exon 1.0 ST Arrays as per the manufacturer’s instructions. Briefly, 5 µg of fragmented biotinylated ssDNA was hybridised for 16 hrs at 45°C, 60 rpm to the array chip. After 16 hrs GeneChips were washed on a FS_450 Fluidics station using the washing script FS450_0001 with buffers and stains supplied with the GeneChip Hybridisation, Wash and Stain Kit from Affymetrix.

### Data Acquisition and Analysis

Data was acquired on a 7G GeneChip Scanner 3000 and.CEL file generation performed using AGCC. Expression Console with Robust Multi-chip Average (RMA) was used initially to extract probe intensity data. This data was used to access Affimetrix supplied PolyA and Eucaryotic Hybridisation controls to confirm consistent processing of the RNA samples. Subsequent statistical analysis was performed in Partek Genomics Suite – Version 6.110801 (Partek Inc. St Louis, MO). The Affymetrix RaEx-1_0-st-v1.r2.dt.core.mps was used to filter probe sets. RMA background correction was applied including pre-background adjustment for GC content and quantile normalization across all chips in the experiment. Probe data was log2 transformed.

### Gene Level Expression Analysis

For gene transcript expression analysis, core probe sets were averaged for each gene. The significance of differences in gene expression between the EAE and healthy control data sets was evaluated by analysis of variance (ANOVA) that compared the controls and normal rats and investigated the effect of different batches. There was no significant batch effect in this study.

Although we are aware that the data may not be distributed normally and that a reduced homogeneity of variance may exist, the alternative non-parametric tests such as the Kruskal Wallis analysis would not produce the ratio of means required to identify statistically significant functional categories and pathways and make predictions about the possible downstream functional effects of fold change for various groups of genes. As a result, the above violations to the assumptions made by this analysis were considered to be adequately compensated for by the robust nature of the analysis. We have produced a list of all the genes that are regulated in EAE, and also a smaller list of the genes that are most highly regulated in MBP-EAE.

### Functional Association Analysis

IPA (Ingenuity® systems, www.ingenuity.com) was used to identify biological functions, gene networks and pathways which were significantly regulated in the spinal cord in EAE compared to healthy controls. In IPA, right-tailed Fisher’s exact test was used to calculate a p-value determining the probability that each function network or pathway assigned to that data set is due to chance alone. Molecules from the data set that met the p<0.05 cut off and were associated with biological functions in the Ingenuity knowledge base were considered for the analysis.

### Downstream Effects Analysis in IPA

The Ingenuity downstream effects analytic were used to identify biological functions which were predicted to be increased or decreased based on the observed gene expression changes in the data-set. The software compared the direction of change for each gene to the causal effects information between genes and biological functions stored in the IPA Knowledge base. Statistical validation of the predictions made were carried out based on the calculation of the regulation z score “which is designed such that data sets composed of randomly chosen perturbed genes with random sign of fold change do not lead to significant results on average” (Ingenuity Downsteam Effects Analysis, whitepaper). Each biological function considered is associated with a number of upstream genes based on annotations derived from the literature and stored in the Ingenuity knowledgebase. The previously observed effects on function of each gene in this set are then used to predict an increase or decrease in the function under the conditions studied. Table 3 shows the biological functions found to be significantly up or down-regulated in MBP induced EAE in the Lewis rat.

### Quantitative RT-PCR Validation of Micro-array Data

Validation of the results obtained from the micro-array experiments was carried out using real time PCR. Five genes (rtCasp1, rtOmg, rtScn1a, rtFas and rtSod2) found to be differentially regulated in EAE in the micro-array gene expression experiments, were selected for validation by RT-PCR. Rat primer pairs were purchased from SA BioSystems (RT^2^qPCR Primer Assay, QIAGEN). Real time PCR analysis was carried on the MyIQ™ real time PCR thermo-cycler and detection system (BioRad Laboratories Inc.) using the RT^2^ QPCR Primer assay according to the manufactures’ protocol. Briefly, 1 µg of total rat spinal cord RNA was treated with QIAGEN Genomic DNA Elimination mix for 5 min at 42?C prior to being reverse transcribed using the kit protocol. RT-PCR conditions were as follows: 95?C, 10 min (Hot Start Taq polymerisation activation step) followed by 40 cycles of 95?C, 15 sec, 60?C, 1 min. The reactions were monitored using SYBR Green containing fluorescein (RT^2^ SYBR Green Fluor qPCR Master mix, QIAGEN). At the end of each PCR run a melting curve analysis was carried out to verify that homogeneous products of equal size had been amplified for each primer pair. The same set of RNA samples that was used for the micro-array experiments was also analysed by RT-PCR and in addition we tested four more independent validation samples from each group, which had been collected and prepared in an identical manner, to test for sampling error. Expression was normalized to rat peptidylprolylisomerase H (rtPpih). Fold changes were calculated using the ΔΔCT method [44] and expressed as fold-change at peak of disease in EAE compared to the expression levels in healthy control Lewis rats (Figure 3).

## Supporting Information

Figure S1 **RNA Virtual Gel Image.** Tissue samples were snap frozen in liquid nitrogen and stored at −80°C prior to total RNA preparation using the QIAGEN RNeasy Lipid tissue kit. RNA quality analysis was carried out on the BioRad Experion automated electrophoresis system. This shows an example of the virtual gel image, illustrate the high quality of samples used. All preparations had RQI values of >9.5.
(DOCX)

Figure S2 **RNA Electropherogram Profile.** Tissue samples were snap frozen in liquid nitrogen and stored at −80°C prior to total RNA preparation using the QIAGEN RNeasy Lipid tissue kit. RNA quality analysis was carried out on the BioRad Experion automated electrophoresis system. This shows an example of the electropherogram profiles illustrating the high quality of samples used. All preparations had RQI values of >9.5.
(DOCX)

Figure S3 **Regulation of the Antigen presentation pathway in MBP-EAE.** This figure shows the upregulation of MHC class I and MHC class II linked pathways of antigen presentation in MBP-EAE.
(TIF)

Figure S4 **Regulation of the T Helper Cell Differentiation in MBP-EAE.** This figure shows that there is upregulation of the pathways of Th0 cell differentiation, especially into Th1 and Th17 cells in MBP-EAE.
(TIF)

Figure S5 **Regulation of Cytotoxic T-lymphocyte mediated apoptosis of target cells in MBP-EAE.** This figure shows that there is upregulation of the pathways involved in T cell cytotoxicity of target cells in MBP-EAE.
(TIF)

Figure S6 **Regulation of Fc gamma –mediated phagocytosis in macrophages and monocytes in MBP-EAE**. This shows that in rats with MBP-EAE there is up-regulation of many of the genes in the pathways leading to phagosome formation after Fc gamma binding.
(TIF)

Figure S7 **Regulation of Interferon signalling in MBP-EAE.** This shows that there is upregulation of many of the genes in the JAK/STAT pathway of interferon signalling in rats with MBP-EAE.
(TIF)

Figure S8 **Regulation of Dendritic cell maturation in MBP-EAE.** This figure shows that many of the genes in the pathway of dendritic cell maturation are upregulated in MBP-EAE, except IL-12 which is downregulated.
(TIF)

Figure S9 **B cell development in MBP-EAE.** This shows that many cell surface receptors involved in the stages of B cell maturation are up- regulated in MBP-EAE.
(TIF)

Figure S10 **Regulation of Trem1 Signalling in MBP-EAE.** This shows that many of the intracellular signalling molecules involved in signalling by the triggering receptor expressed on myeloid cells 1 (TREM1) are upregulated in MBP-EAE. This pathway is involved in adaptive and innate immunity.
(TIF)

Figure S11 **Regulation of Communication between innate and adaptive immune cells in MBP-EAE.** This shows that many signalling molecules involved in this pathway are upregulated except for IL12 which is down regulated.
(JPG)

Figure S12 **Regulation of the Complement pathway in MBP-EAE.** This shows that the classical pathway and the alternate pathway but not the common terminal pathway are regulated in MBP-EAE.
(TIF)

Figure S13 **Regulation of genes in the pathway entitled Pathogenesis of Multiple Sclerosis.** This is a pathway of chemokine receptors. The receptors CCR1, CCR5, CXCR3, CCL5, CXCL9 and CXCL10 are up-regulated in MBP-EAE.
(TIF)

Figure S14 **Regulation of Synaptic long term potentiation in MBP-EAE.** This shows that this pathway of signalling in response to glutamate is down-regulated in MBP-EAE.
(TIF)

Figure S15 **Regulation of Dopamine-DARPP32 feedback in cAMP signalling in MBP-EAE.** This shows that this pathway of signalling in response to dopamine is down-regulated in MBP-EAE.
(TIF)

Figure S16 **Regulation of the Amyotrophic Lateral Sclerosis pathway in MBP-EAE.** This pathway of intra-cellular signalling that leads to cell death and degeneration in response to glutamate is down-regulated in MBP-EAE.
(TIF)

Figure S17 **Regulation of Creb signalling in neurons in MBP-EAE.** This shows that this pathway of intracellular signalling leading to expression of cAMP response element-binding (Creb) is down-regulated in MBP-EAE.
(TIF)

Figure S18 **Regulation Endothelin-1 signalling in MBP-EAE.** This shows that this pathway of intracellular signalling in response to endothelin 1 (ET1) is downregulated in MBP-EAE.
(TIF)

Figure S19 **Regulation the Biosynthesis of steroids in MBP-EAE.** This shows that the squalene pathway involved in the bio-synthesis of steroids is down-regulated in MBP-EAE.
(TIF)

Figure S20 **Regulation Taurine/hypotaurine metabolism in MBP-EAE.** This shows downregulation of elements of the pathway that leads to synthesis of taurine and hypotaurine from cysteine in MBP-EAE.
(TIF)

Table S1 (XLSX)

Table S2 (XLSX)
