# Supplementary figures and images for: Gene Expression in the Spinal Cord in Female Lewis Rats with Experimental Autoimmune Encephalomyelitis Induced with Myelin Basic Protein
Source: PLoS One. 2012 Nov 6;7(11):e48555. doi: 10.1371/journal.pone.0048555 (PMC3491034; doi:10.1371/journal.pone.0048555)

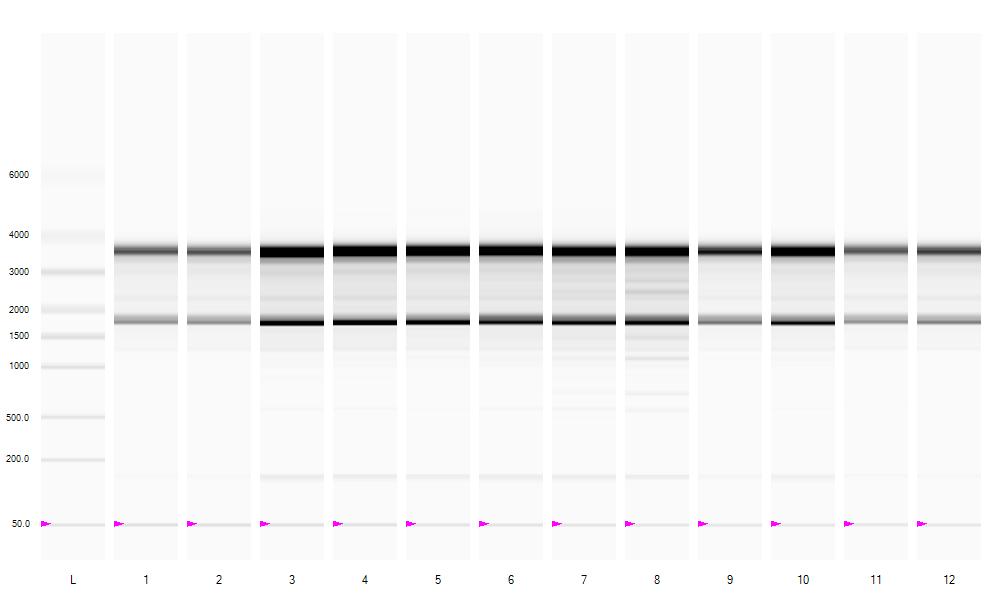

Supplement: Figure S1 — RNA Virtual Gel Image. Tissue samples were snap frozen in liquid nitrogen and stored at −80°C prior to total RNA preparation using the QIAGEN RNeasy Lipid tissue kit. RNA quality analysis was carried out on the BioRad Experion automated electrophoresis system. This shows an example of the virtual gel image, illustrate the high quality of samples used. All preparations had RQI values of >9.5. (DOCX) [file pone.0048555.s001.docx]

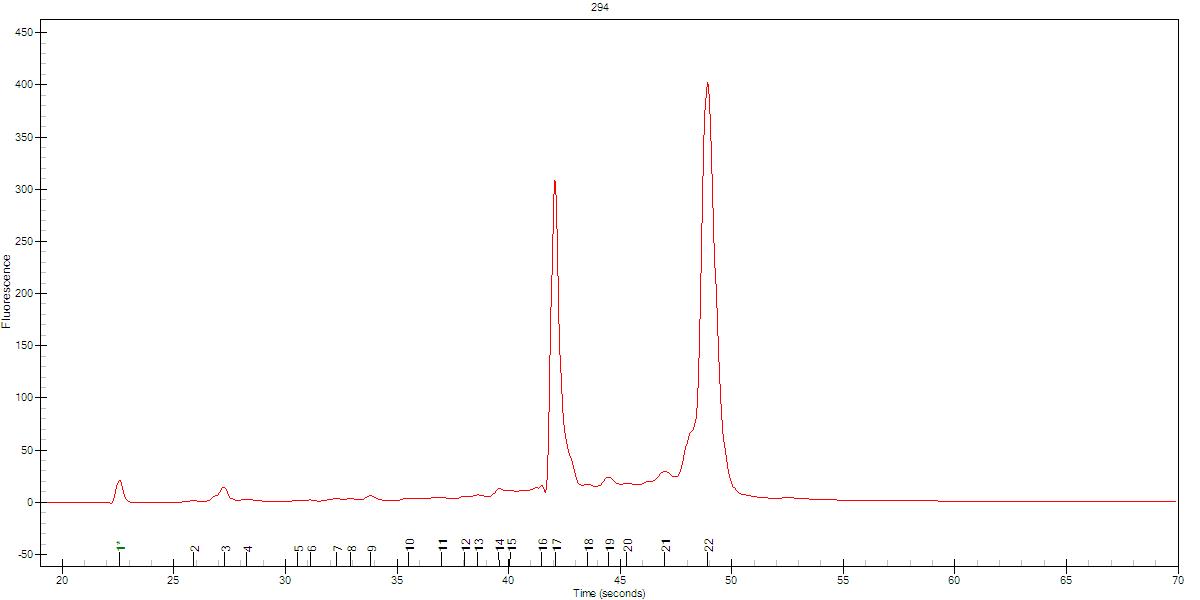


Time (seconds)

Fluorescence

Supplement: Figure S2 — RNA Electropherogram Profile. Tissue samples were snap frozen in liquid nitrogen and stored at −80°C prior to total RNA preparation using the QIAGEN RNeasy Lipid tissue kit. RNA quality analysis was carried out on the BioRad Experion automated electrophoresis system. This shows an example of the electropherogram profiles illustrating the high quality of samples used. All preparations had RQI values of >9.5. (DOCX) [file pone.0048555.s002.docx]

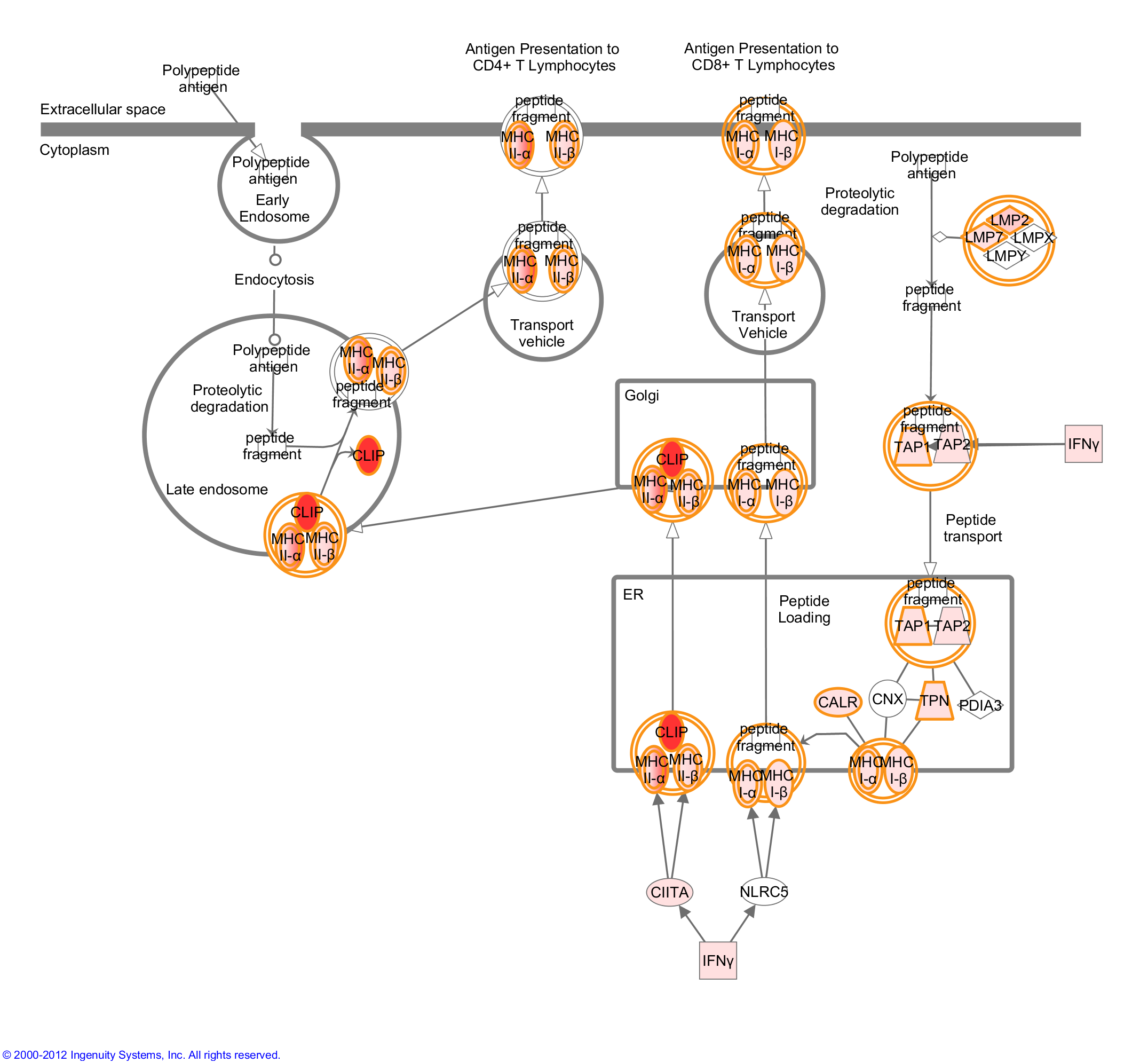

Supplement: Figure S3 — Regulation of the Antigen presentation pathway in MBP-EAE. This figure shows the upregulation of MHC class I and MHC class II linked pathways of antigen presentation in MBP-EAE. (TIF) [file pone.0048555.s003.tif]

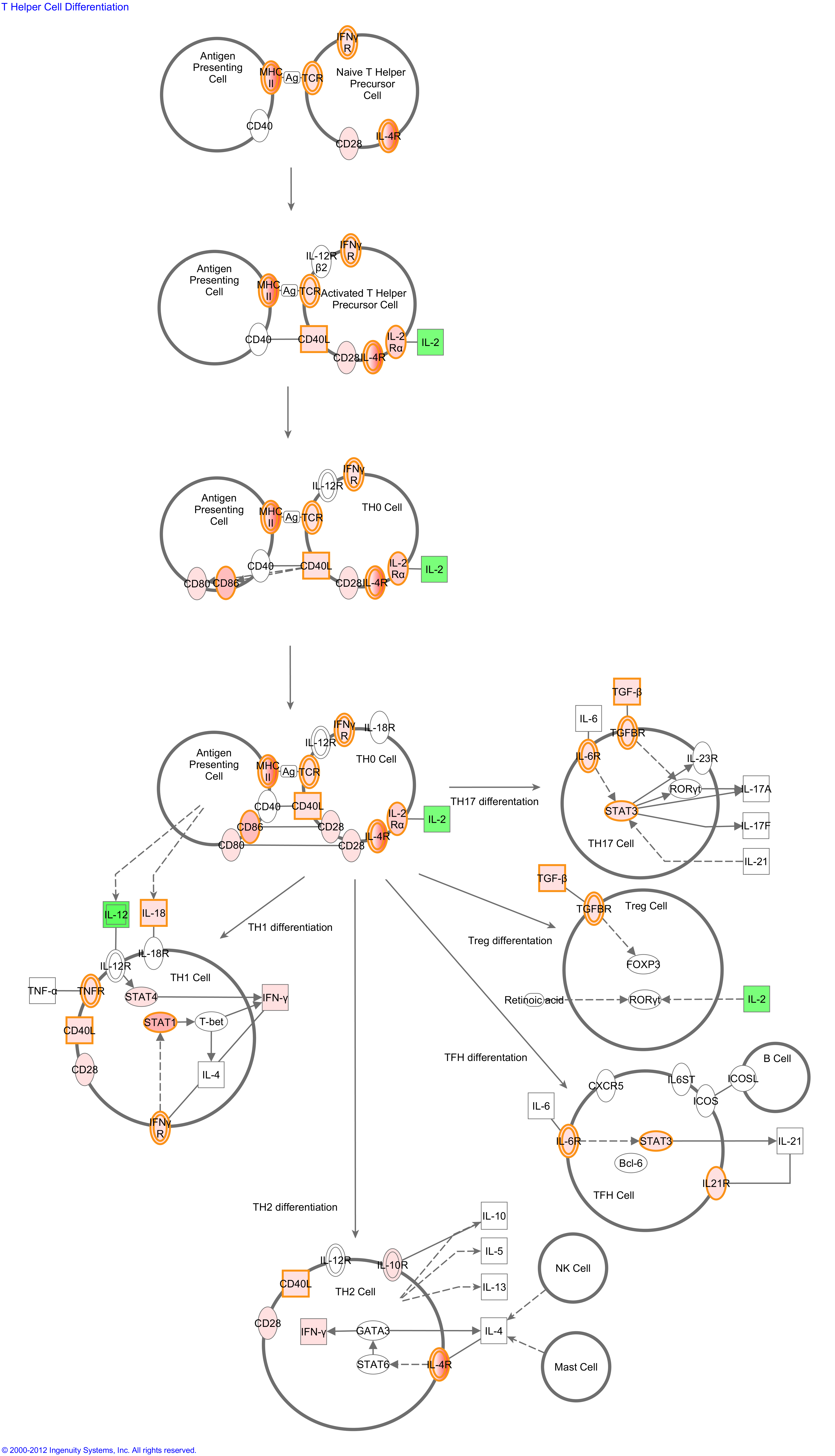

Supplement: Figure S4 — Regulation of the T Helper Cell Differentiation in MBP-EAE. This figure shows that there is upregulation of the pathways of Th0 cell differentiation, especially into Th1 and Th17 cells in MBP-EAE. (TIF) [file pone.0048555.s004.tif]

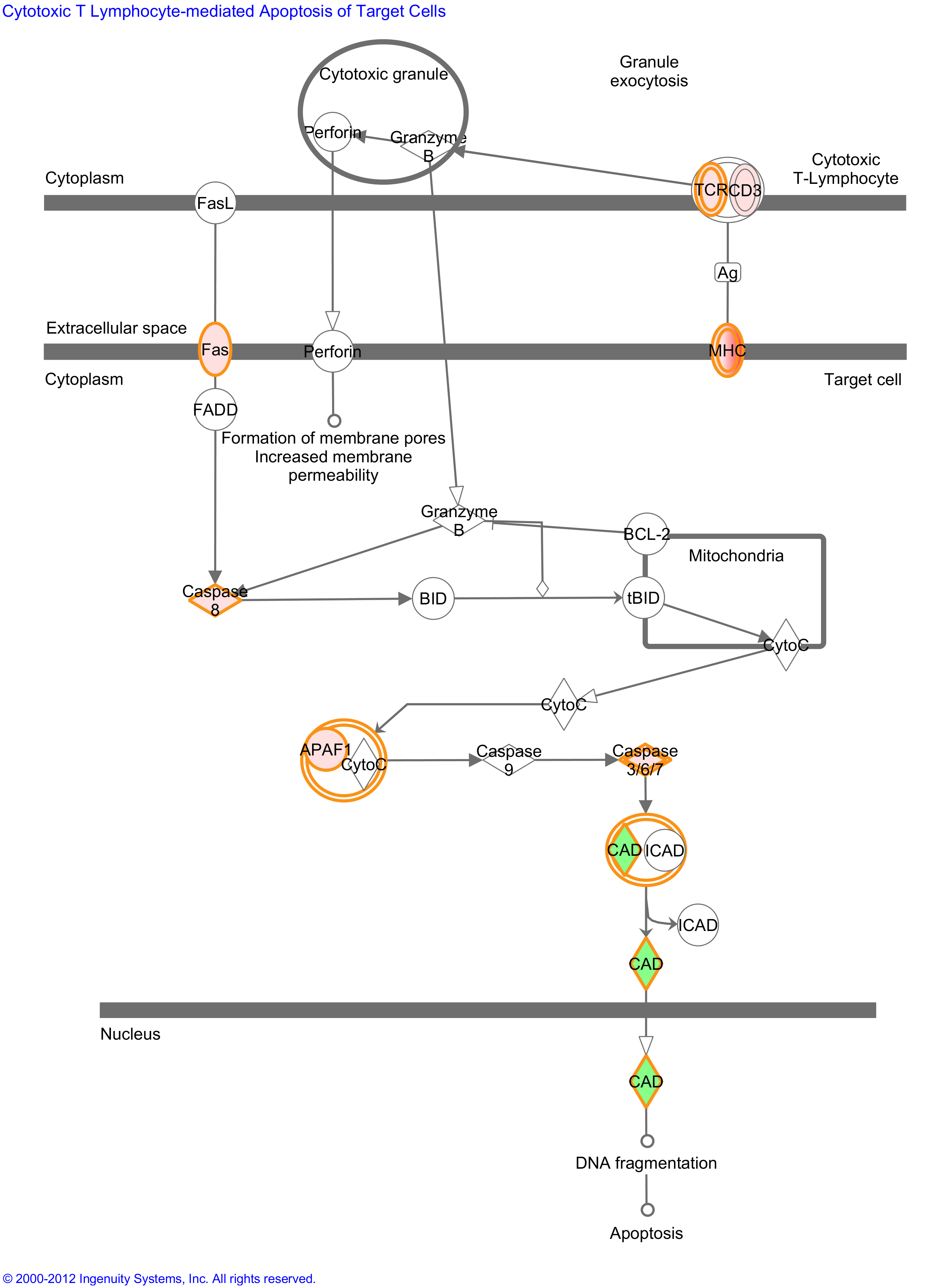

Supplement: Figure S5 — Regulation of Cytotoxic T-lymphocyte mediated apoptosis of target cells in MBP-EAE. This figure shows that there is upregulation of the pathways involved in T cell cytotoxicity of target cells in MBP-EAE. (TIF) [file pone.0048555.s005.tif]

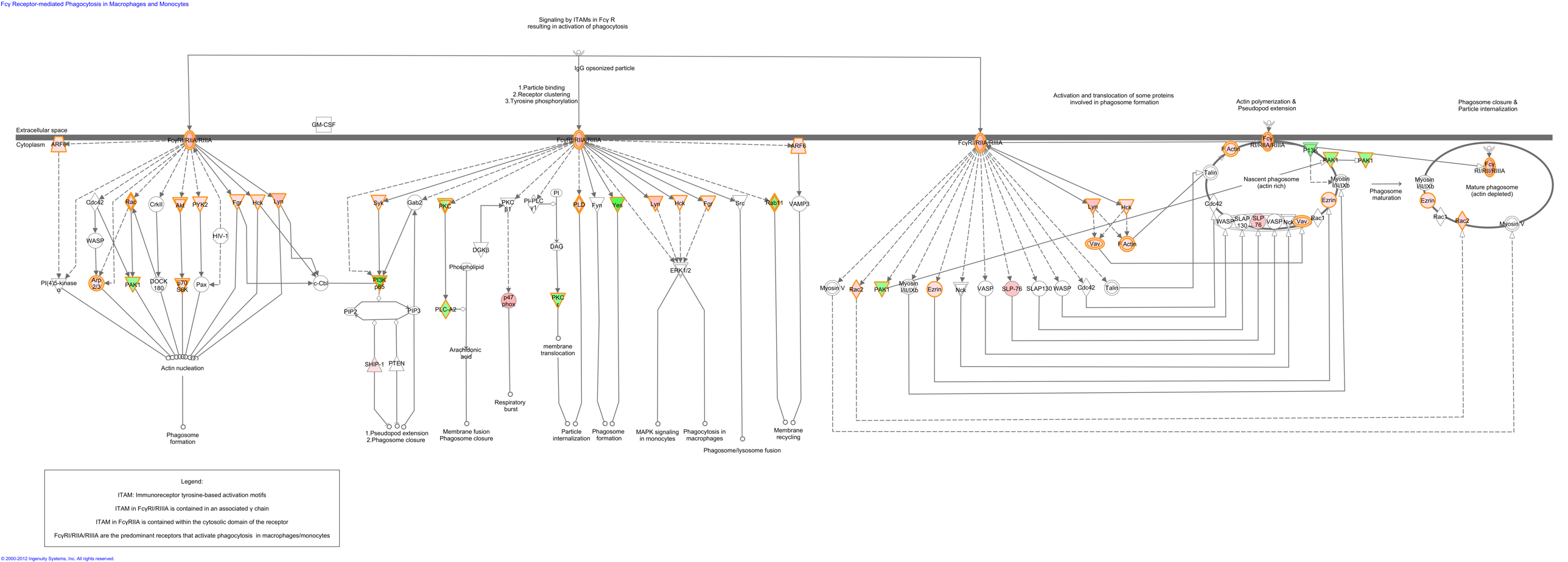

Supplement: Figure S6 — Regulation of Fc gamma –mediated phagocytosis in macrophages and monocytes in MBP-EAE. This shows that in rats with MBP-EAE there is up-regulation of many of the genes in the pathways leading to phagosome formation after Fc gamma binding. (TIF) [file pone.0048555.s006.tif]

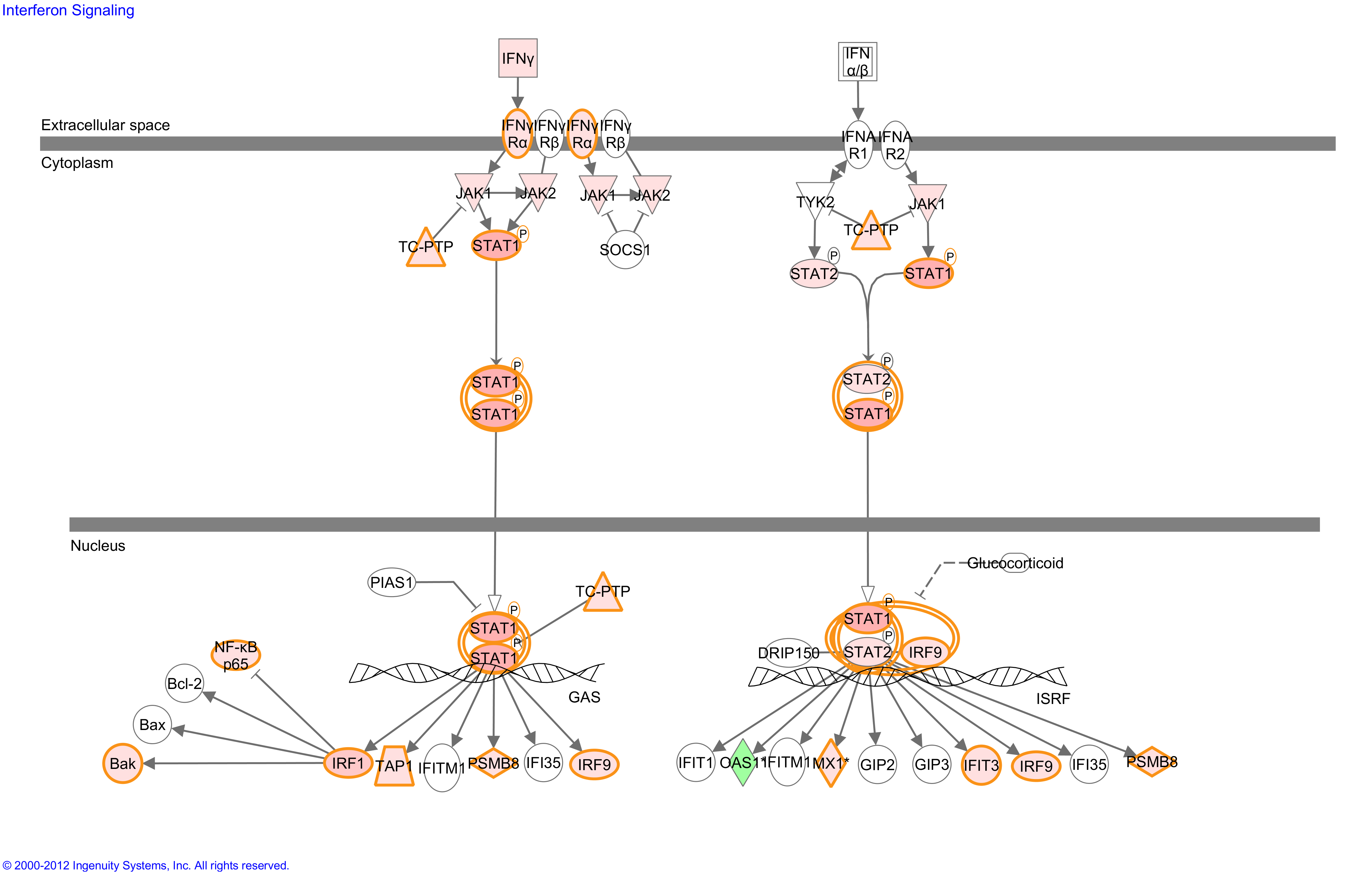

Supplement: Figure S7 — Regulation of Interferon signalling in MBP-EAE. This shows that there is upregulation of many of the genes in the JAK/STAT pathway of interferon signalling in rats with MBP-EAE. (TIF) [file pone.0048555.s007.tif]

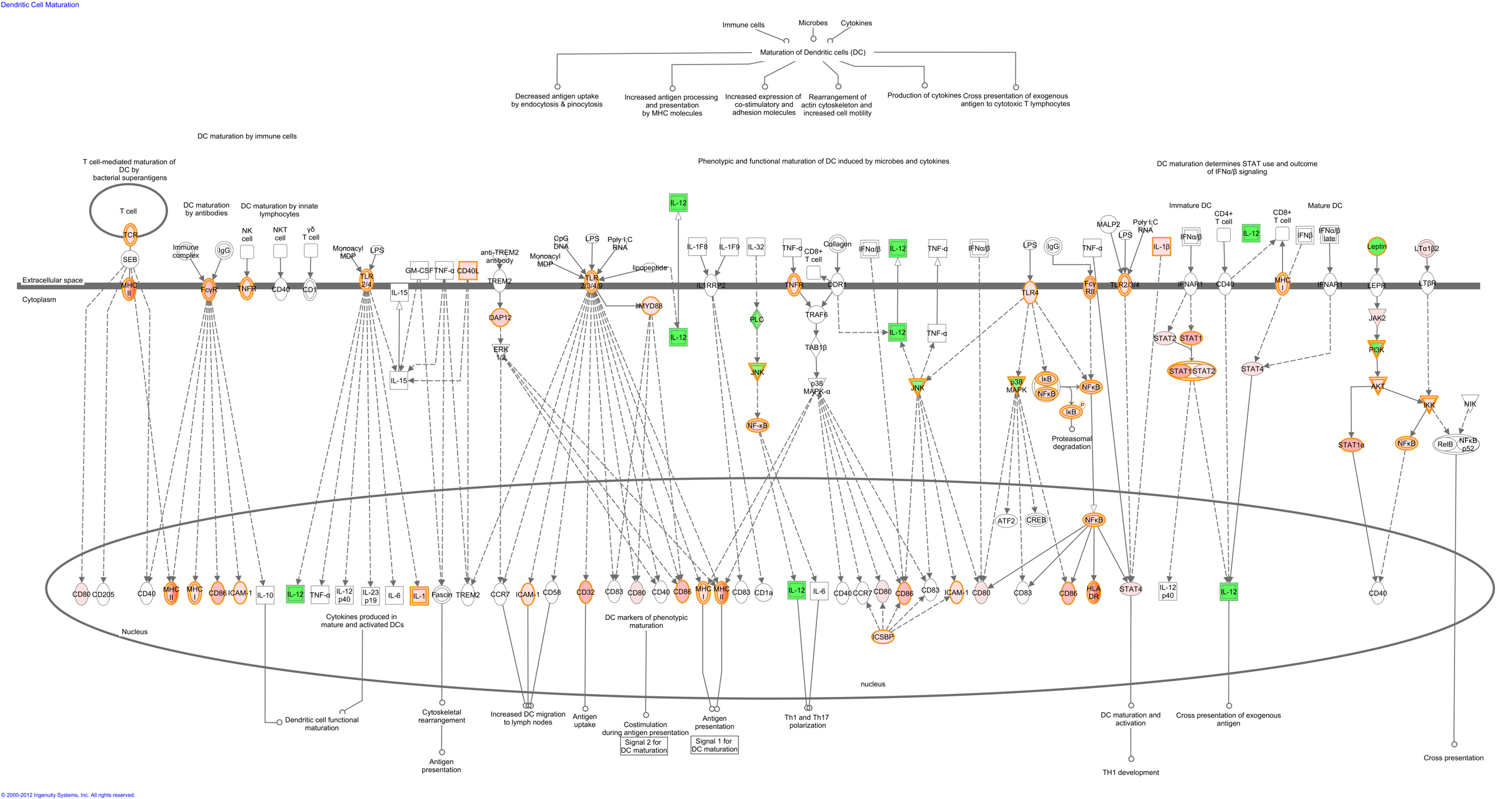

Supplement: Figure S8 — Regulation of Dendritic cell maturation in MBP-EAE. This figure shows that many of the genes in the pathway of dendritic cell maturation are upregulated in MBP-EAE, except IL-12 which is downregulated. (TIF) [file pone.0048555.s008.tif]

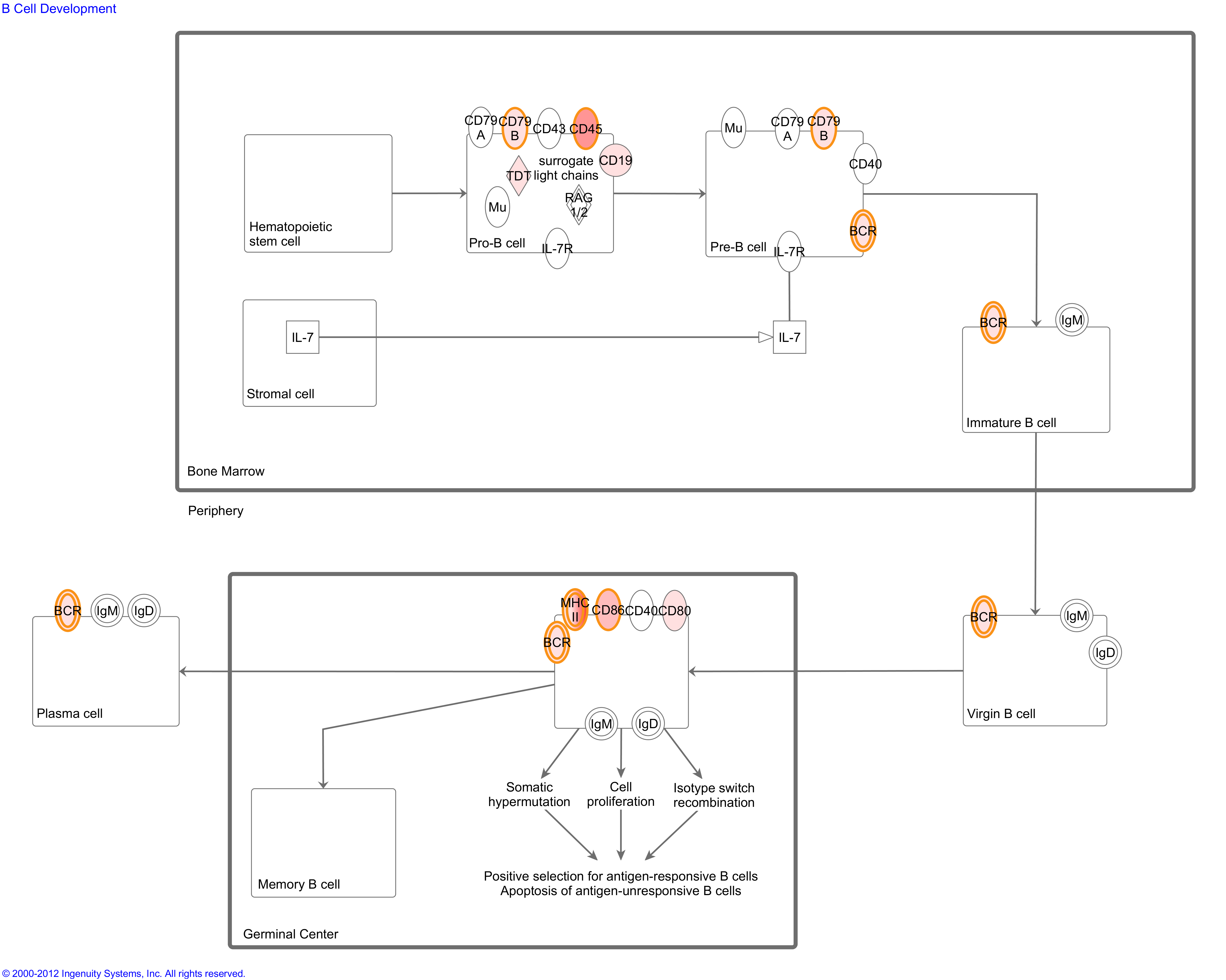

Supplement: Figure S9 — B cell development in MBP-EAE. This shows that many cell surface receptors involved in the stages of B cell maturation are up- regulated in MBP-EAE. (TIF) [file pone.0048555.s009.tif]

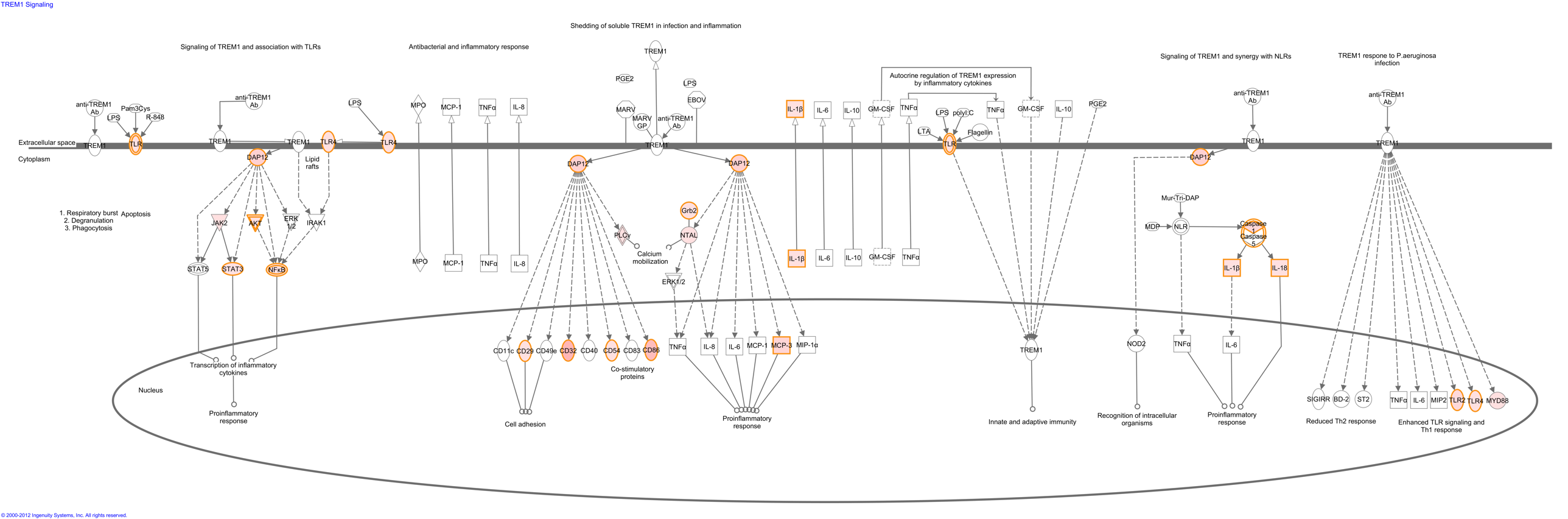

Supplement: Figure S10 — Regulation of Trem1 Signalling in MBP-EAE. This shows that many of the intracellular signalling molecules involved in signalling by the triggering receptor expressed on myeloid cells 1 (TREM1) are upregulated in MBP-EAE. This pathway is involved in adaptive and innate immunity. (TIF) [file pone.0048555.s010.tif]

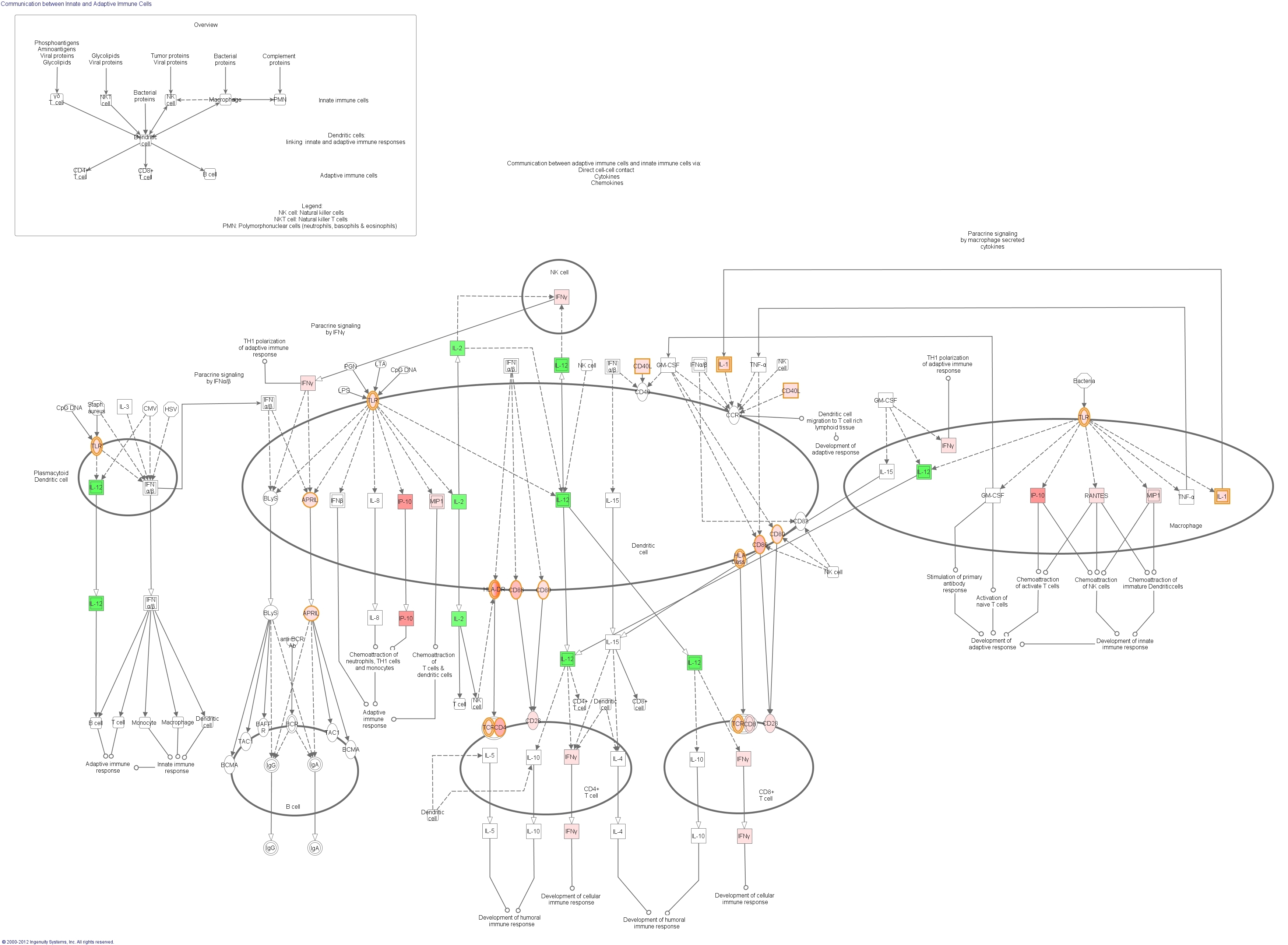

Supplement: Figure S11 — Regulation of Communication between innate and adaptive immune cells in MBP-EAE. This shows that many signalling molecules involved in this pathway are upregulated except for IL12 which is down regulated. (JPG) [file pone.0048555.s011.jpg]

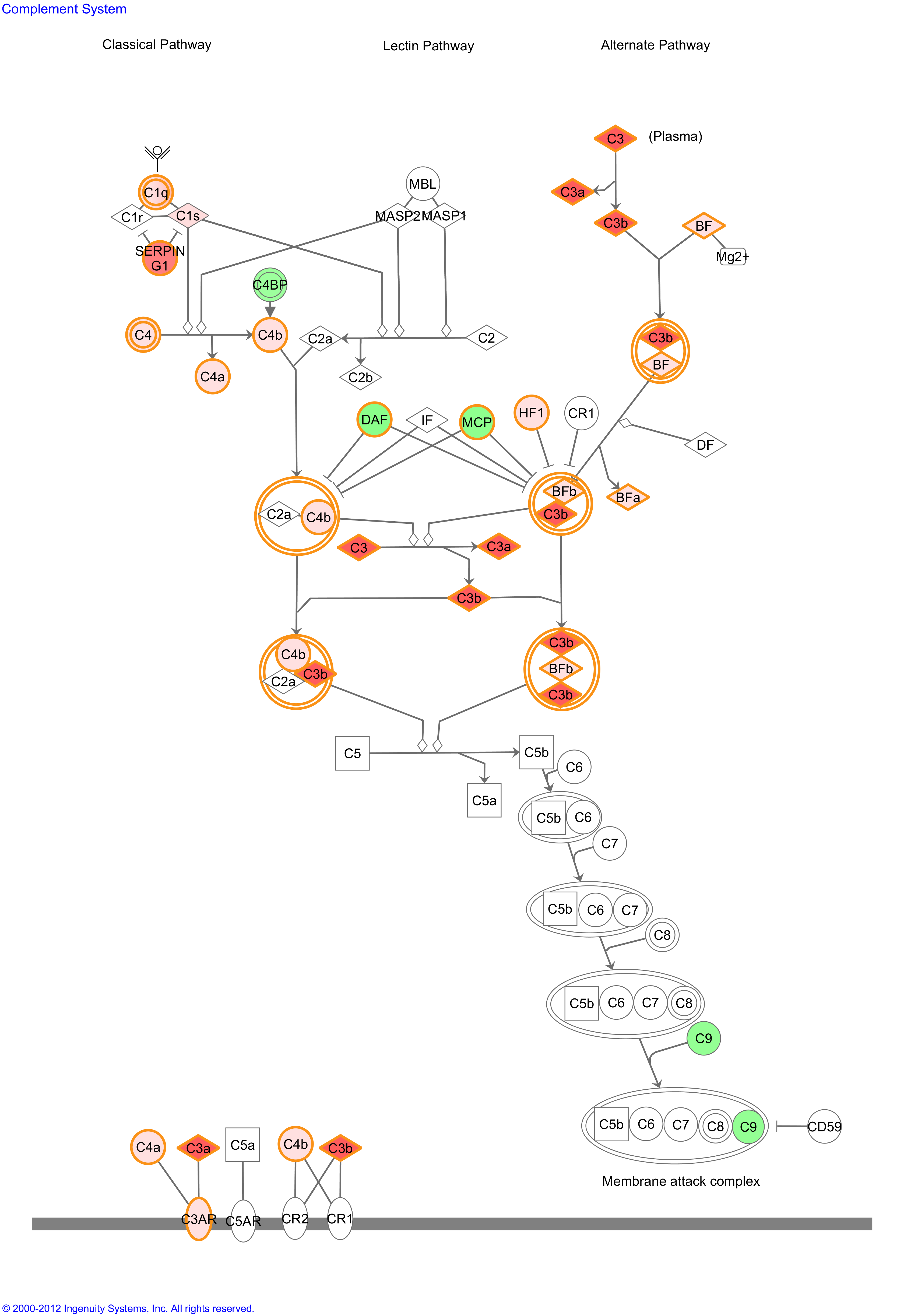

Supplement: Figure S12 — Regulation of the Complement pathway in MBP-EAE. This shows that the classical pathway and the alternate pathway but not the common terminal pathway are regulated in MBP-EAE. (TIF) [file pone.0048555.s012.tif]

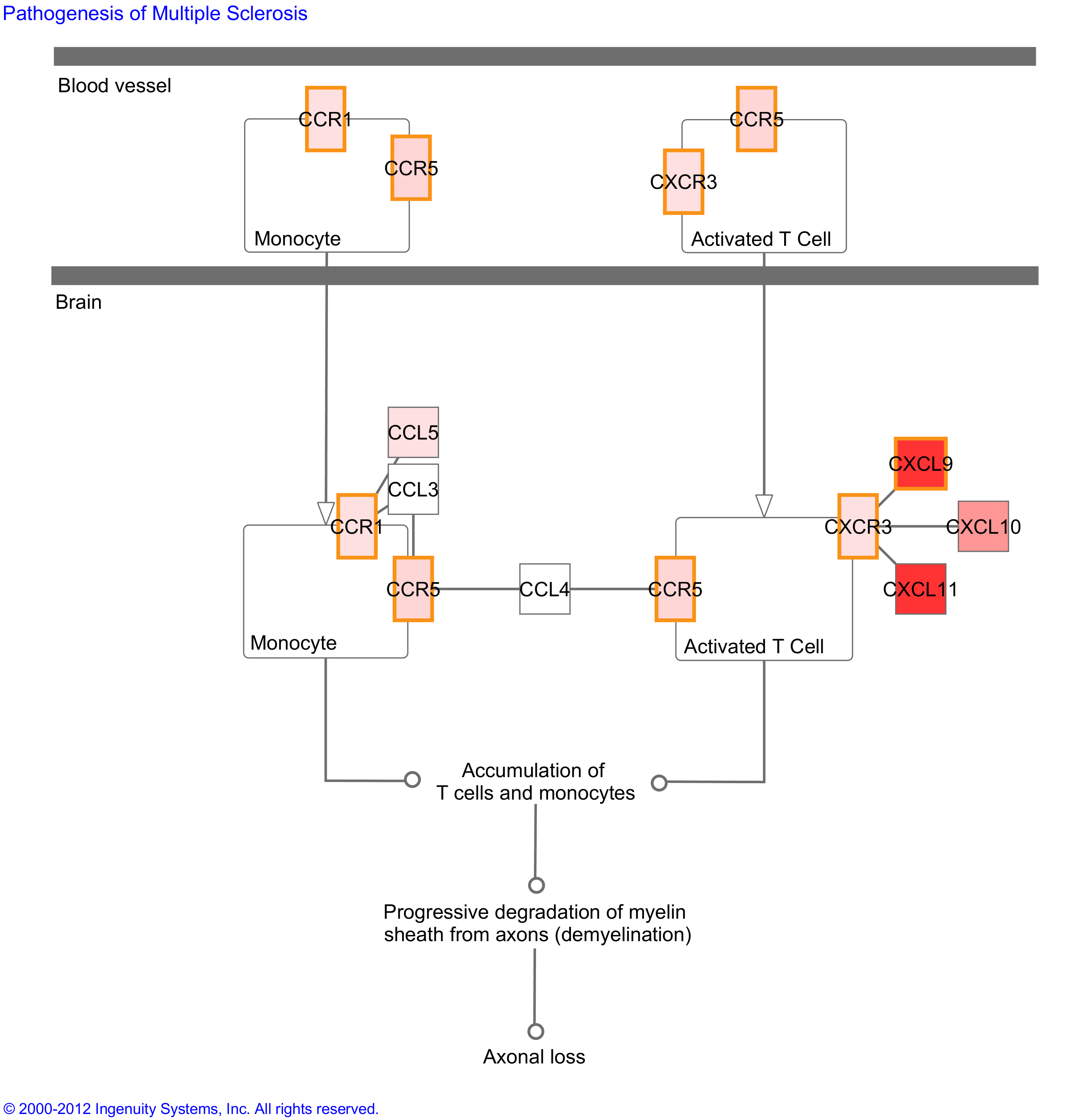

Supplement: Figure S13 — Regulation of genes in the pathway entitled Pathogenesis of Multiple Sclerosis. This is a pathway of chemokine receptors. The receptors CCR1, CCR5, CXCR3, CCL5, CXCL9 and CXCL10 are up-regulated in MBP-EAE. (TIF) [file pone.0048555.s013.tif]

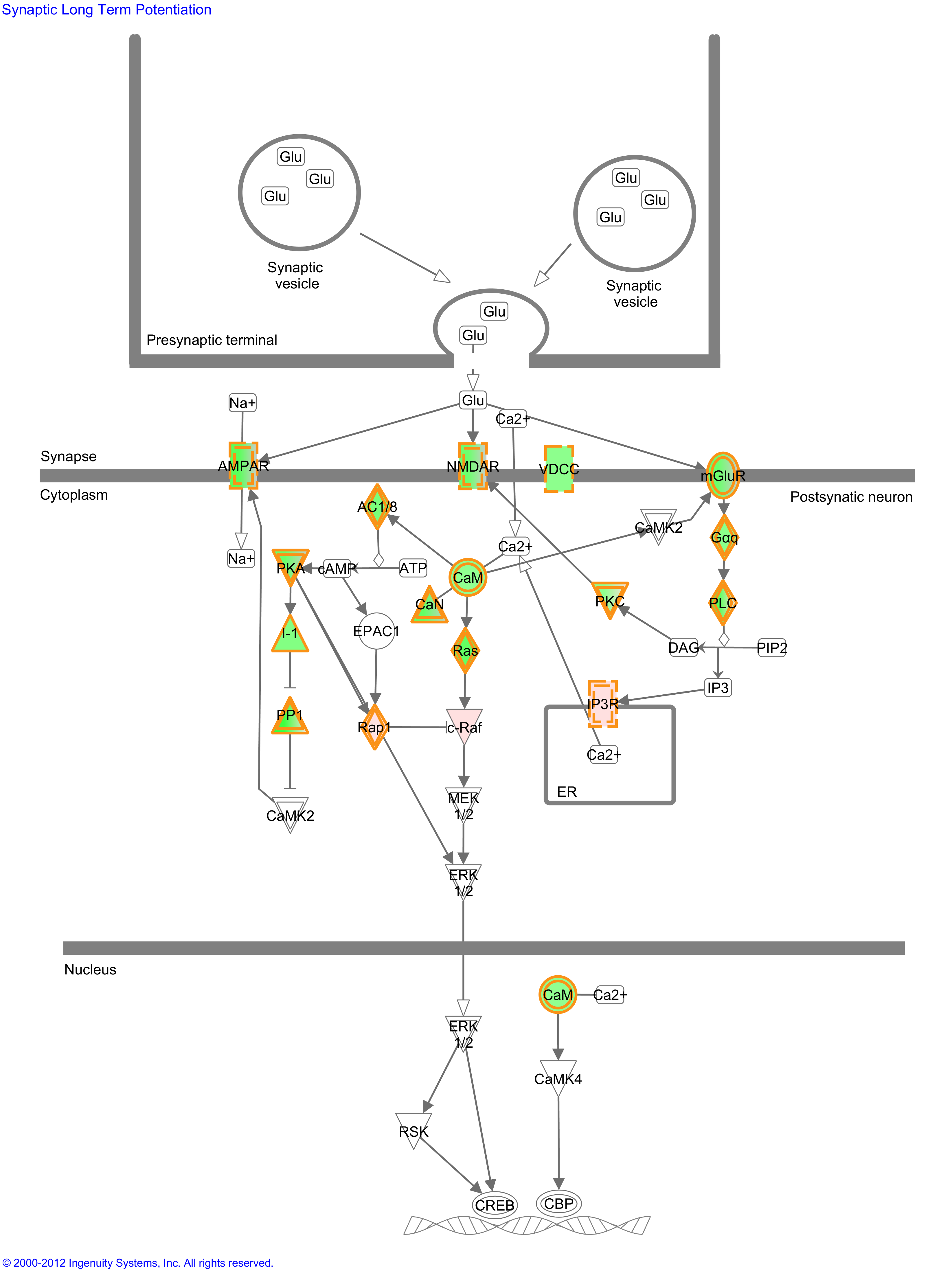

Supplement: Figure S14 — Regulation of Synaptic long term potentiation in MBP-EAE. This shows that this pathway of signalling in response to glutamate is down-regulated in MBP-EAE. (TIF) [file pone.0048555.s014.tif]

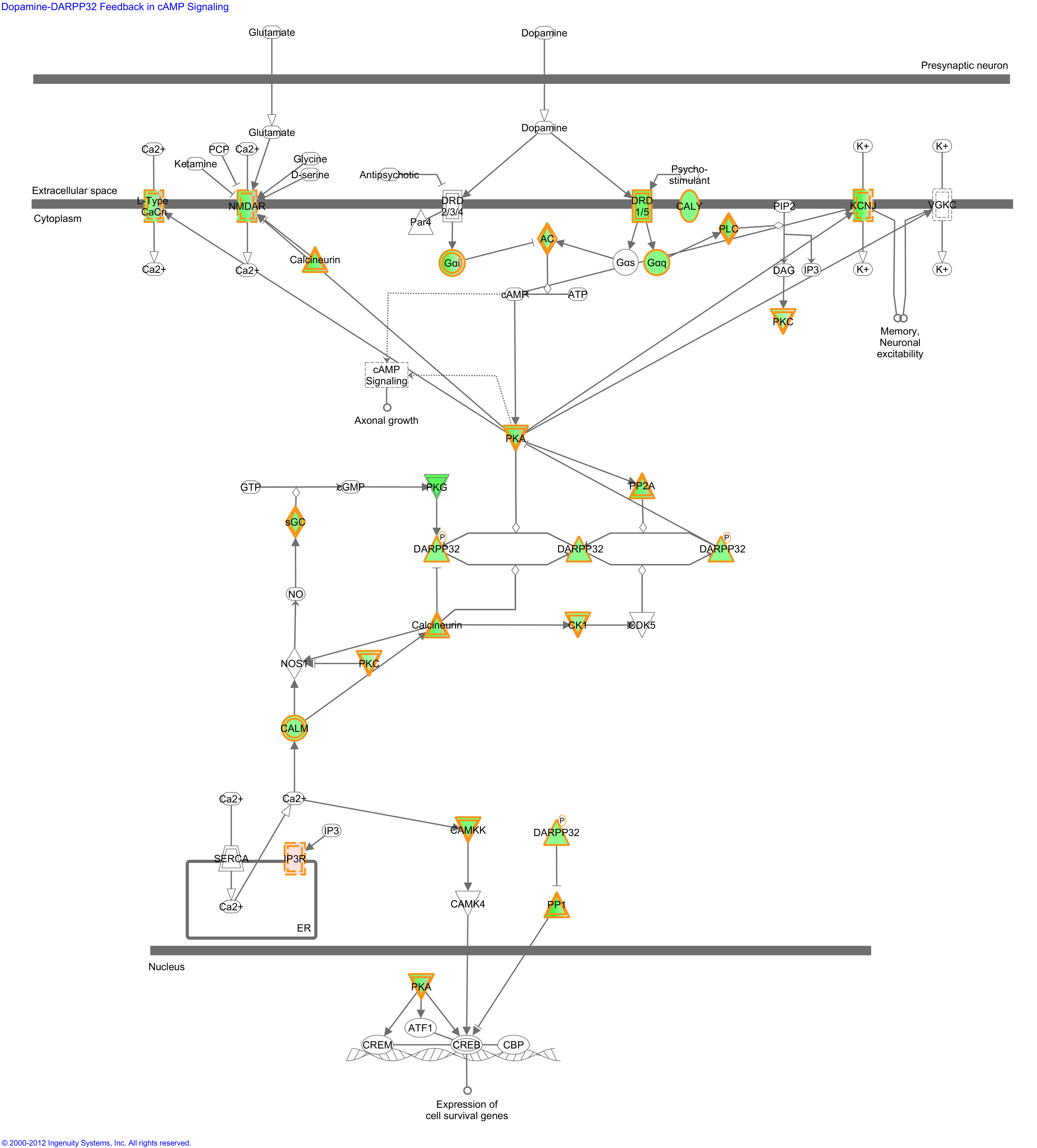

Supplement: Figure S15 — Regulation of Dopamine-DARPP32 feedback in cAMP signalling in MBP-EAE. This shows that this pathway of signalling in response to dopamine is down-regulated in MBP-EAE. (TIF) [file pone.0048555.s015.tif]

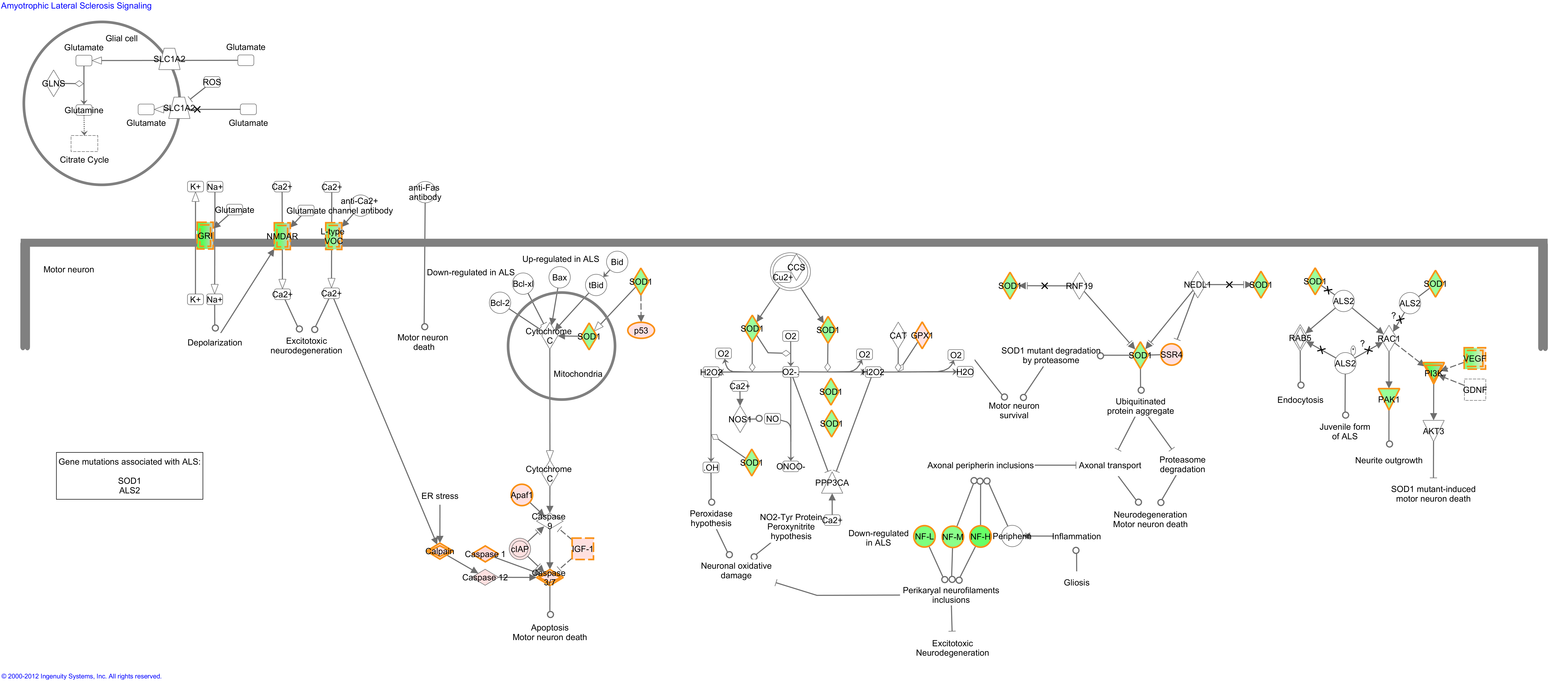

Supplement: Figure S16 — Regulation of the Amyotrophic Lateral Sclerosis pathway in MBP-EAE. This pathway of intra-cellular signalling that leads to cell death and degeneration in response to glutamate is down-regulated in MBP-EAE. (TIF) [file pone.0048555.s016.tif]

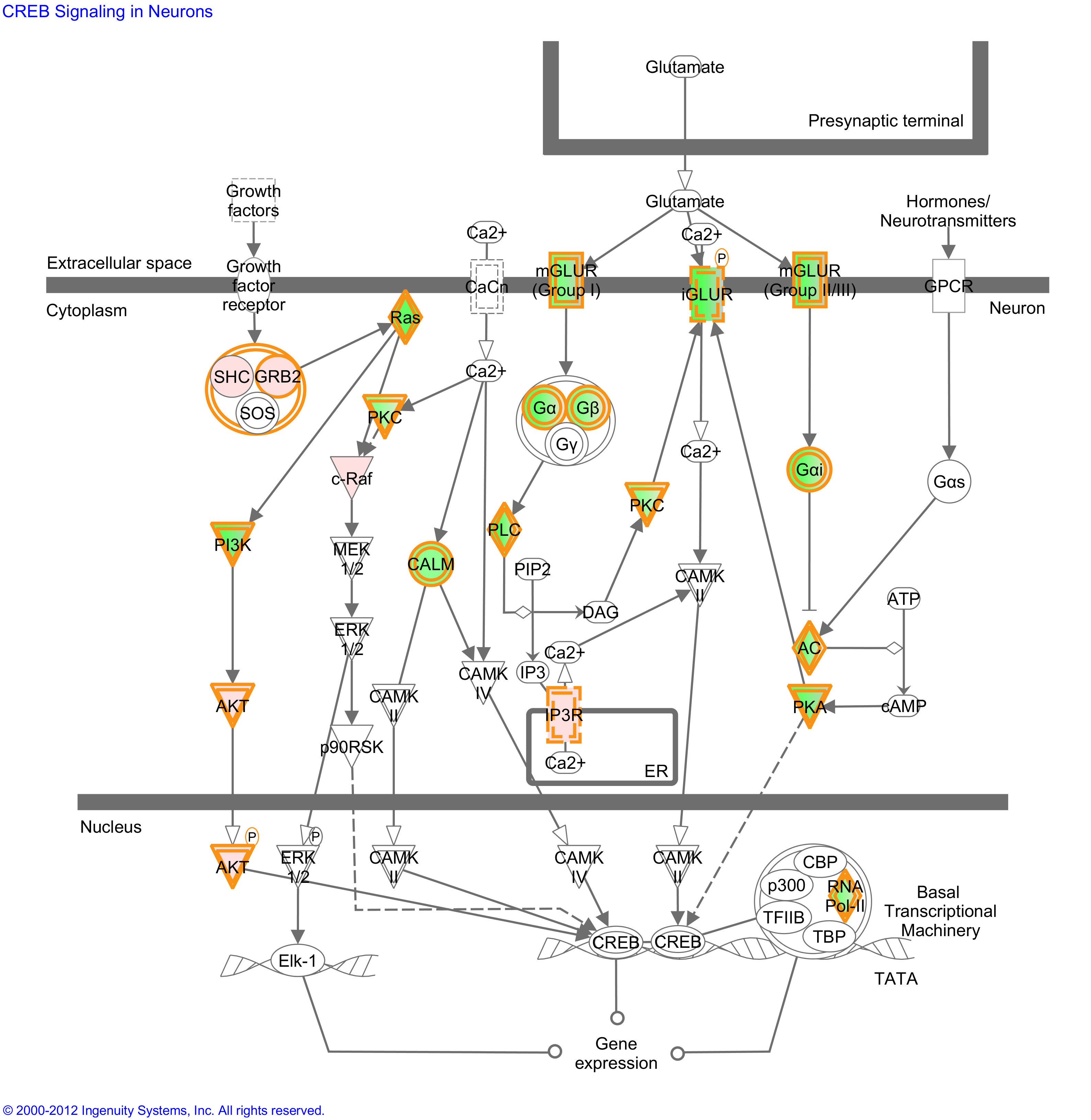

Supplement: Figure S17 — Regulation of Creb signalling in neurons in MBP-EAE. This shows that this pathway of intracellular signalling leading to expression of cAMP response element-binding (Creb) is down-regulated in MBP-EAE. (TIF) [file pone.0048555.s017.tif]

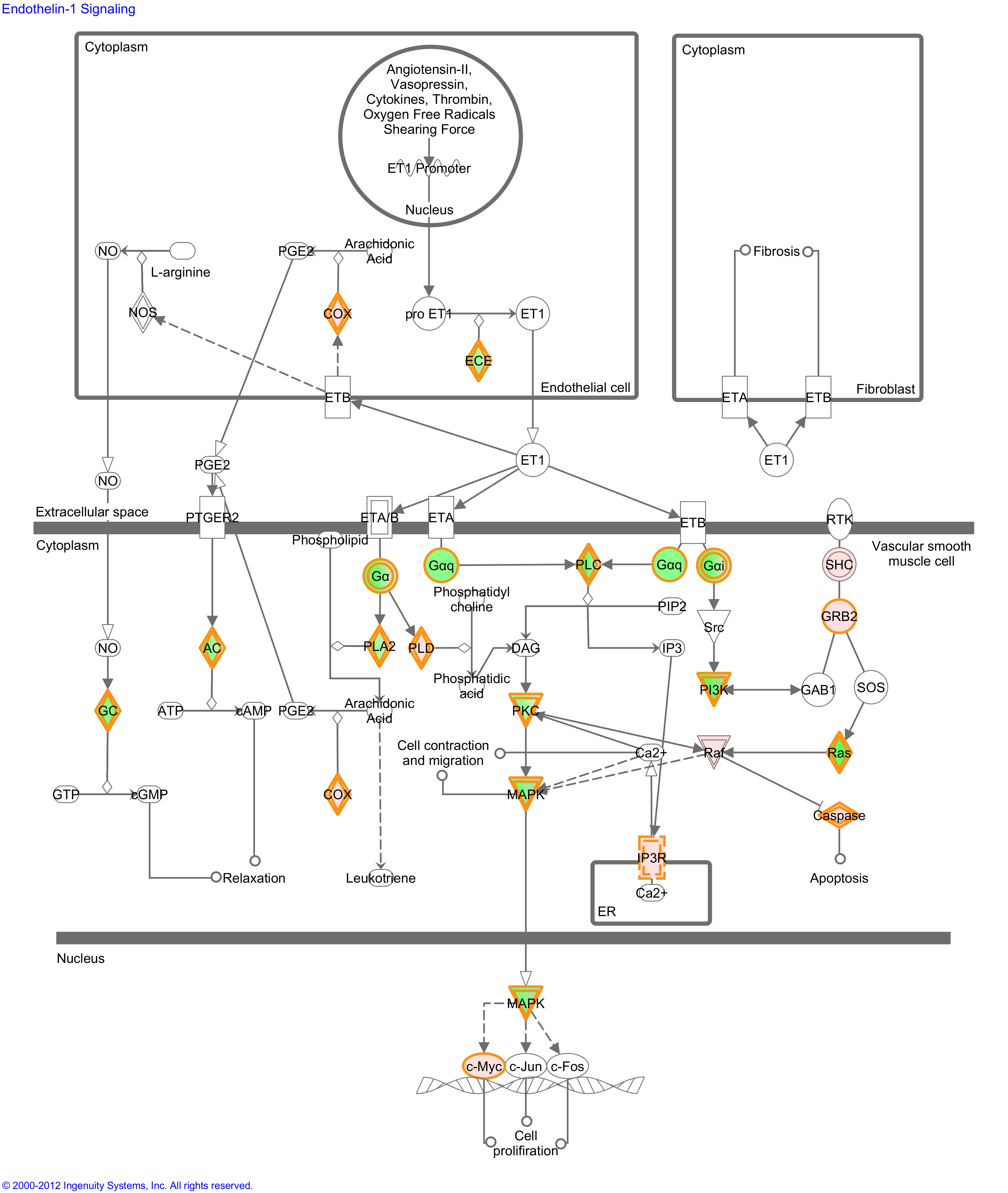

Supplement: Figure S18 — Regulation Endothelin-1 signalling in MBP-EAE. This shows that this pathway of intracellular signalling in response to endothelin 1 (ET1) is downregulated in MBP-EAE. (TIF) [file pone.0048555.s018.tif]

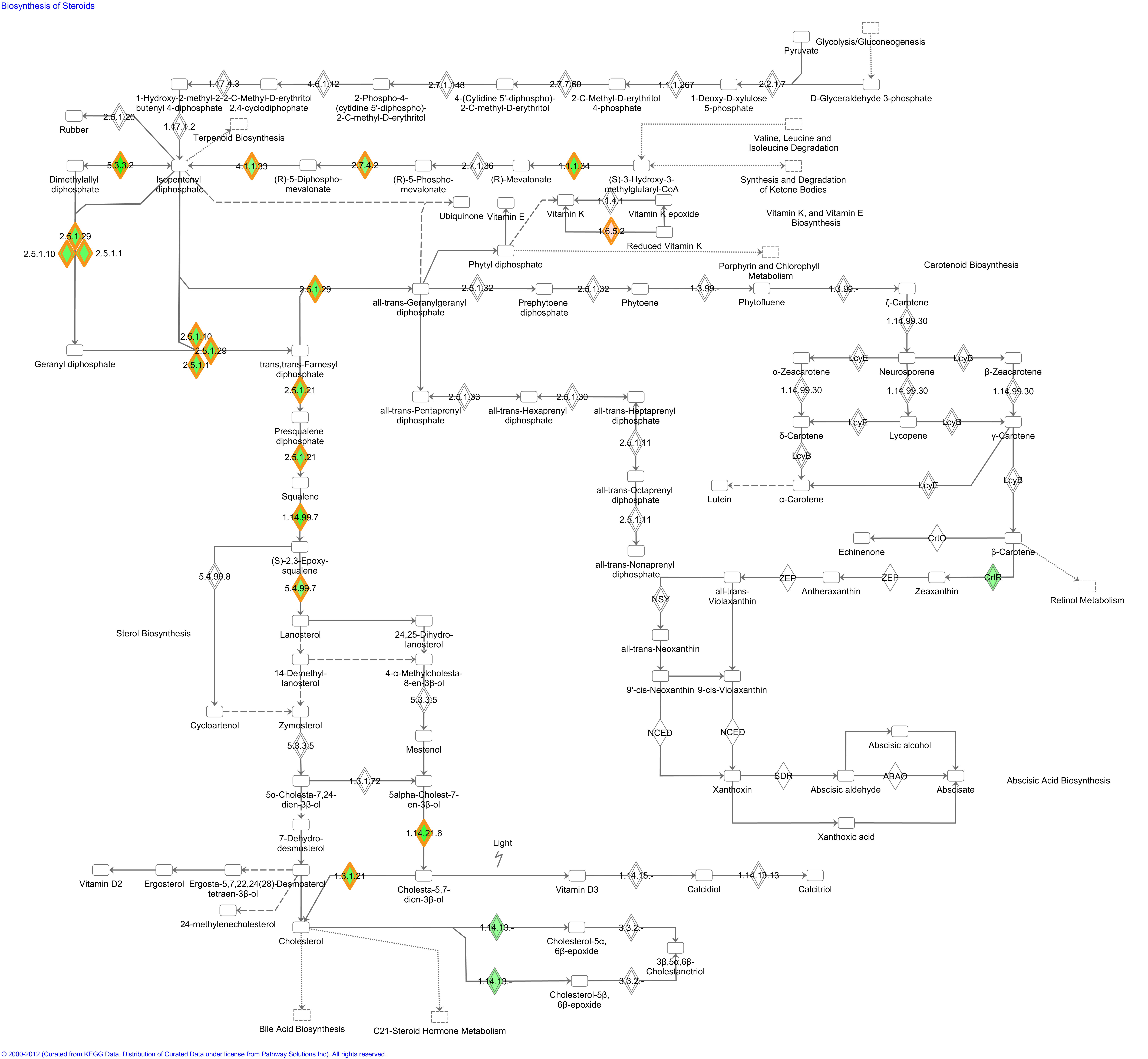

Supplement: Figure S19 — Regulation the Biosynthesis of steroids in MBP-EAE. This shows that the squalene pathway involved in the bio-synthesis of steroids is down-regulated in MBP-EAE. (TIF) [file pone.0048555.s019.tif]

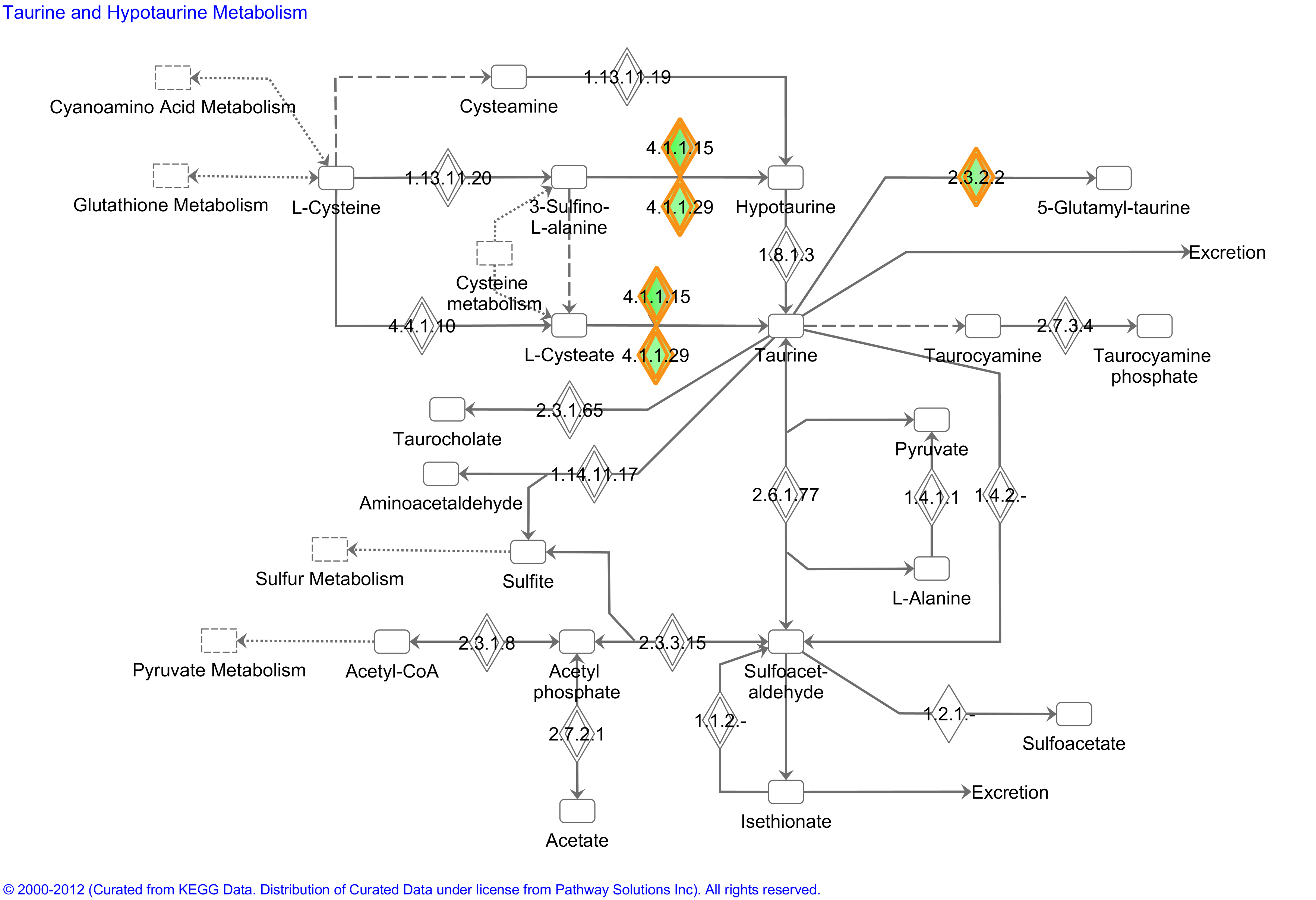

Supplement: Figure S20 — Regulation Taurine/hypotaurine metabolism in MBP-EAE. This shows downregulation of elements of the pathway that leads to synthesis of taurine and hypotaurine from cysteine in MBP-EAE. (TIF) [file pone.0048555.s020.tif]
